# Isolation, characterization and in vitro anti-salmonellal activity of compounds from stem bark extract of *Canarium schweinfurthii*

**DOI:** 10.1186/s12906-020-03100-5

**Published:** 2020-10-19

**Authors:** Jean Baptiste SOKOUDJOU, Olubunmi ATOLANI, Guy Sedar Singor NJATENG, Afsar KHAN, Cyrille Ngoufack TAGOUSOP, André Nehemie BITOMBO, Norbert KODJIO, Donatien GATSING

**Affiliations:** 1 Research Unit of Microbiology and Antimicrobial substances, Faculty of Science, University of Dschang, P.O. Box 67, Dschang, Cameroon; 2 Natural Products Chemistry Laboratory, Department of Chemistry, COMSATS University Islamabad, Abbottabad Campus-22060, Islamabad, Pakistan; 3 Department of Chemistry, Faculty of Physical Sciences, University of Ilorin, P.M.B, Ilorin, 1515 Nigeria; 4 Department of Basic Scientific Studies, University Institute of Technology, University of Ngaoundere, P.O.Box 455, Ngaoundere, Cameroon; 5 Department of Organic Chemistry, Faculty of Science, University of Yaoundé I, P.O. Box 812, Yaoundé, Cameroon

**Keywords:** Ethnomedicine, Salmonellosis, *Canarium schweinfurthii*, Natural substances

## Abstract

**Background:**

Bacteria belonging to the *Salmonella* genus are major concern for health, as they are widely reported in many cases of food poisoning. The use of antibiotics remains a main stream control strategy for avian salmonellosis as well as typhoid and paratyphoid fevers in humans. Due to the growing awareness about drug resistance and toxicities, the use of antibiotics is being discouraged in many countries whilst advocating potent benign alternatives such as phyto-based medicine. The objective of this work was to isolate, characterise the bioactive compounds of *Canarium schweinfurthii*; and evaluate their anti-salmonellal activity.

**Methods:**

The hydro-ethanolic extract of *Canarium schweinfurthii* was fractionated and tested for their anti-salmonellal activity. The most active fractions (i.e. chloroform and ethyl acetate partition fractions) were then explored for their phytochemical constituents. Fractionation on normal phase silica gel column chromatography and size exclusion chromatography on Sephadex LH-20 led to the isolation of four compounds (maniladiol, scopoletin, ethyl gallate and gallic acid) reported for the first time in *Canarium schweinfurthii*.

**Results:**

Result indicated that scopoletin and gallic acid had greater activity than the crude extracts and partition fractions. Among the isolated compounds, scopoletin showed the highest inhibitory activity with a MIC of 16 μg/ml against *Salmonella* Typhimurium and *Salmonella* Enteritidis.

**Conclusions:**

The overall results of this study indicates that the hydro-ethanolic extract as well as some of isolated compounds have interesting anti-salmonellal activities that could be further explored for the development of potent therapy for salmonellosis. Furthermore, the study adds credence to the folkloric applications of the plant.

## Background

*Salmonella* is a major source of food-borne illness in humans and a major cause of morbidity, mortality and economic loss both in the poultry and human health sectors. The disease caused by bacteria belonging to *Salmonella* genus is often called salmonellosis. This pathology remains one of the limiting factors in the development of poultry farming especially in developing countries of Asia and Africa [1] because it causes huge direct and indirect losses [2]. The genus *Salmonella* is very diverse and today it is composed of more than 2500 serotypes, many of which cause enteric diseases in humans and animals. Many serotypes of *Salmonella* can infect chickens and some serotypes are well adapted although, *Salmonella* Gallinarum and *Salmonella* Pullorum cannot be transmitted to human. However, some serotypes can infect both poultry and human and among these serotypes *Salmonella* Enteritidis and *Salmonella* Typhimurium are more prevalent in chickens and notable in human disease outbreaks. These serotypes are most commonly implicated in the human *Salmonella* infections [3, 4]. The poultry is considered one of the main sources of *Salmonella* human infection usually through poorly cooked foods [5–9] and foodstuffs of avian origin [10]. *Salmonella* infection represents a considerable burden in both developing and developed countries. Ubiquitous non-typhoidal *Salmonella* (NTS) which includes *Salmonella* Enteritidis and *Salmonella* Typhimurium annually cause more than 93.8 million illnesses and 155,000 deaths each year [11]. *Salmonella* Enteritidis and *Salmonella* Typhimurium, both NTS are the most frequently occurring serotypes from poultry causing infection in human [3]. Similarly, each year worldwide, typhoidal serotypes among which *Salmonella* Typhi and *Salmonella* Paratyphi, cause approximately 22 million cases of typhoid and 216,500 deaths [12].

Resistance of *Salmonella* to commonly used antimicrobial agents is increasing both in the veterinary and public health sectors and has emerged as a global health challenge. Several *Salmonella* serotypes are multidrug resistant, and there is evidence of the spread of these strains from animals to humans. Antimicrobial resistance in NTS is considered one of the major public health threats related with food-animal production, as well as the poultry production chain and poultry meat, which is an additional concern in the management of salmonellosis [13]. Many authors [14–17] have reported that several strains of *Salmonella* isolated from chicken have shown resistance to many antibiotics commonly used in human medicine and some of these strains have been found in humans [14]. Moreover, antibiotic residues in poultry products intended for consumption may lead to hypersensitivity or poisoning in consumers. Due to the growing awareness of resistance issues, the use of antibiotics is strongly discouraged in many countries whilst encouraging the use of plants as a better alternative due to their diverse nature of bioactive principles [18–20]. The large majority of salmonellosis in humans is carried by foodstuffs; mainly those of avian origin [10, 20, 21], therefore controlling avian salmonellosis by using plant could significantly reduce the prevalence of human gastroenteritis [20]. Several studies have focused on medicinal plants as new control strategies for human salmonellosis [22, 23] or avian salmonellosis [24–28]. But, to our knowledge, no phytomedicine has yet been formulated to control avian salmonellosis. *Canarium schweinfurthii* Engl. (Burseraceae), is a tree with a cylindrical bole, native to tropical West Africa and grows to about 50 m high [29]. This plant is mainly found in equatorial forest regions from Cameroon, Central African Republic, Gabon to Congo [30] and is used in folk medicine for the treatment of various diseases including malaria, diarrhea and Typhoid fever [31, 32]. Previous studies of Sokoudjou et al. [20, 28] showed that the hydroethanolic extracts of *Canarium schweinfurthii* were active both in vitro and in vivo against several serotypes of *Salmonella*. The objective of this work was to isolate, characterise the bioactive compounds of *Canarium schweinfurthii*; and evaluate their anti-salmonellal activity.

## Methods

### General experiment

Reagents which include ammonium cerium sulphate, were of analytical grade. Solvents were distilled before being used (St Louis, MO, USA). Thin Layer Chromatography (TLC) was performed on pre-coated silica gel with thickness 0.20 mm 60 F_254_ plates (MerckKGaA, Germany) and viewed under the UV light (254 and 365 nm). NMR analyses which included ^1^H NMR, ^13^C NMR, DEPT 90, DEPT 135, 2D NMR (COSY, HSQC), NOESY and ROESY were performed using deuterated solvents (Acétone-*d*_6,_ CD_3_OD and/or CDCl_3_) on 400 MHz NMR (Ascend™ 400, Bruker) with TMS as internal reference. ESI-MS spectra of the compounds were recorded on a Bruker-Ion Trap MS (MicroTOF-Q mass spectrometer, Bruker) using the positive mode.

### Plant collection, identification and extraction

*Canarium schweinfurthii* stem bark was harvested in West region of Cameroon and identified at the National Herbarium at Yaoundé-Cameroon, where a voucher specimen was deposited under the reference Number 16929/SRF/Cam. The air-dried plant material (3 Kg) was powdered and macerated at room temperature with 12 L of ethanol-water system (50/50, v/v). After 48 h, the mixture was filtrated using Whatman №1 filter paper. The filtrate was evaporated using a Rotary evaporator (Büchi R200) at reduced pressure to afford the crude extract (265 g, 8.8%).

We needed no permission to collect the sample since *Canarium schweinfurthii* is not a protected species in Cameroon.

### Fractionation and isolation of bioactive compounds of *Canarium schweinfurthii*

**Fig. 1:**
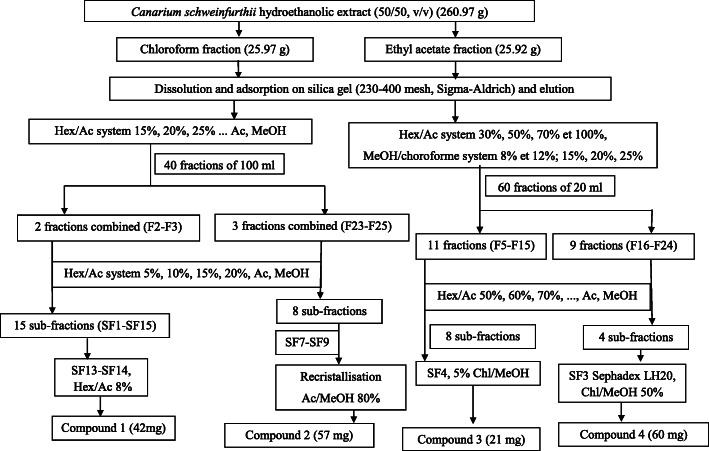
Flow chart for the isolation of compounds from the hydroethanolic extract of *Canarium schweinfurthii*

The profiling of the hydro-ethanolic extract of *Canarium schweinfurthii* on TLC plates with several solvent systems showed no promising separation. In order to facilitate isolation, 260 g of extract was dissolved in distilled water (700 mL) and successively extracted with hexane (500 mL × 2), chloroform (500 mL × 2), ethyl acetate (500 mL × 2) and *n*-butanol (500 mL × 2) yielding respectively 5.56 g, 25.97 g, 25.92 g and 90.89 g of fractions after evaporation to dryness. These partition fractions were explored for their antibacterial activity and only the most active fractions were selected for the isolation of bioactive principles. Figure 1 below shows the protocol for isolating the bioactive principles of *Canarium schweinfurthii*.

Part of Chloroform fraction (23 g) was subjected to silica gel column chromatography using *n*-hexane-EtOAc (85:15 → 00:100) and MeOH, gradient elution. 40 sub-fractions of 100 mL each were collected and combined on the basis of their TLC profiles to give 5 fractions: A (1–3), B (4–12), C (13–22), D (23–25) and E (25–40). Sub-fraction A (4.5 g) was purified on silica gel column chromatography eluted with *n*-hexane-EtOAc (95:5 → 80:20) to give compound **1** (42 mg). The purification of sub-fraction D (4 g) on silica gel column chromatography using *n*-hexane-EtOAc (70:30 → 20:80) afforded compound **2** (57 mg) which was recrystallized in EtOAc-MeOH (20:80).

Part of EtOAc fraction (23 g) was also subjected to silica gel column chromatography eluted with a gradient of *n*-hexane-EtOAc (70:30 → 00:100) and chloroform-MeOH (92:5 → 75:25) to afford 60 sub-fractions of 20 mL which were combined to four sub-fractions: F (1–4), G (5–15) H (16–24), I (25–60) on the basis of their TLC profile. Sub-fraction G (3.5 g) was purified on silica gel column chromatography using *n*-hexane-EtOAc (50:50 → 00:100) to yield compound **3** (21 mg) while purification of sub-fraction H (2.6 g) on sephadex LH-20 column eluted with chloroform-methanol (50:50) afforded compound **4** (60 mg). The structures of the isolated compounds were elucidated by combining various techniques comprising 1D Nuclear Magnetic Resonance (NMR):^1^H NMR, ^13^C-NMR, DEPT 90, DEPT 135 and 2D NMR (COSY, HSQC), NOESY and ROESY as well as Mass Spectrometry analysis (TOF-ESI-MS). The data of the established structures were compared with those existing in literature.

### Anti-salmonellal assay

#### Chemicals for anti-salmonellal assay

Ciprofloxacin (BDH Chemicals, England) and oxytetracyclin (BDH Chemicals, England) were used as reference antibiotics. P-iodonitrotetrazolium chloride (Sigma-Aldrich, Germany) was used as microbial growth indicator.

#### Test bacteria and culture media

Three clinical isolates (*Salmonella* Typhi, *Salmonella* Enteritidis and *Salmonella* Typhimurium from Pasteur Center, Yaoundé-Cameroon) and one bacterial strain (*Salmonella* Typhi ATCC6539 from American Type Culture Collection) were used for antimicrobial evaluation. The culture media used were Salmonella-Shigella Agar (SSA from HiMedia Laboratories, India) and Mueller Hinton Broth (MHB from HiMedia Laboratories, India).

#### Determination of minimal inhibitory concentrations (MICs) and minimal bactericidal concentrations (MBCs)

The MIC values of the fractions obtained from partition and compounds from *Canarium schweinfurthii* were determined in 96-wells microplates using rapid INT colorimetric assay [33, 34]. Briefly, each sample was dissolved in 5% Dimethyl-sulfoxide (DMSO)/MHB. The obtained solution was then added to 100 μL of MHB, and followed by two-fold serial dilution. Then 100 μL of inoculum (1.5 × 10^6^ CFU/mL) prepared in MHB were added to each well except the negative control wells. The plates were covered with a sterile plate sealer and incubated at 37 °C for 18 h. The wells containing either MHB or MHB and 100 μL of inoculum served as control. After the incubation, 40 μL of INT (0.2 mg/mL) was added to each well and plates were re-incubated at 37 °C for 30 min, and the MIC of each sample was recorded. MIC was defined as the lowest concentration of the sample that prevented change in colour and exhibited complete inhibition of microbial growth. The MBC was determined by adding 50 μL aliquots of the preparations, which did not show any growth after incubation during MIC assays, to 150 μL of MHB. These preparations were then incubated at 37 °C for 48 h. The MBC was recorded as the lowest concentration of test sample which did not produce a colour change after addition of INT as previously described. The tests were performed in triplicates.

## Results

The yield and physical appearance of each partition fraction of *Canarium schweinfurthii* extract are as shown below (Table 1).

**Table 1:** Yield and physical appearance of each partition fraction of *Canarium schweinfurthii* stem barks extracts

Partitioned fractions	Yields (%)	Physical characteristics	
		Color	Physical appearance
**Hexane fraction**	2	Green	Oily
**Chloroform fraction**	10	Dark brown	Oily
**Ethylacetate fraction**	10	Brown	Solid
**n-butanol fraction**	34	Blackish	Cristalline powder
**Residual fraction**	38	Blackish	Sticky semi-solid (Syrup)

### Characterization of isolated compounds

**Fig. 2:**
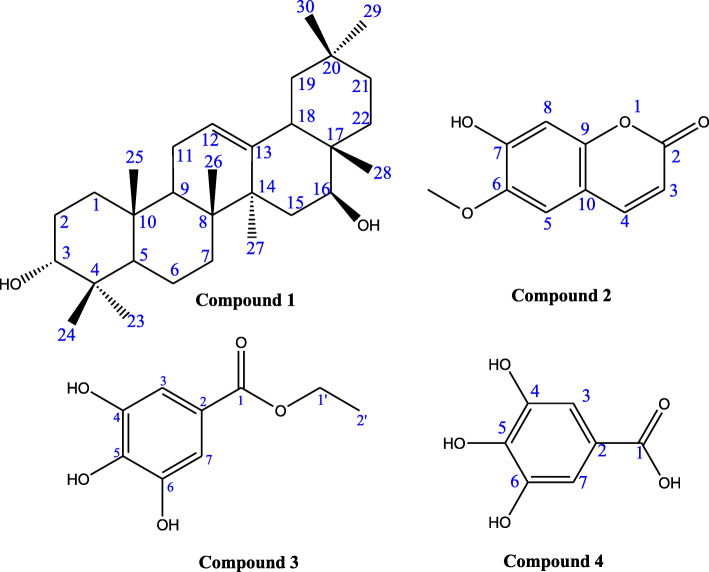
Chemical structures of isolated compounds from *Canarium schweinfurthii* stem barks extract

The four compounds isolated and characterized from the stem bark extract of *Canarium schweinfurthii* are as depicted in Fig. 2.

Compound 1: Maniladiol (42 mg) white solid, soluble in methanol, with molecular weight 442 calculated for C_30_H_50_O_2_ (ESI-MS: m/z 465.1 [M + Na]).

Compound 2: Scopoletin (57 mg) yellowish crystals, soluble in acetone, with molecular weight 192 calculated for C_10_H_8_O_4_ (ESI-MS: m/z 214.9 [M + Na]).

Compound 3: Ethyl gallate (21 mg) white solid, soluble in methanol, with molecular weight 198 calculated for C_9_H_10_O_5_ (ESI-MS: m/z 221.0 [M + Na]).

Compound 4: Gallic acid (60 mg) white solid, soluble in methanol, with molecular weight 170 calculated for C_7_H_6_O_5_ (ESI-MS: m/z 193.1 [M + Na]).

The ^1^H-NMR and ^13^C-NMR data of isolated compounds are presented in the Tables 2, 3, 4 and 5.

**Table 2:** ^1^H-NMR and ^13^C-NMR of compound 1

Compound 1			Maniladiol, Quijano et al. [35]	
Positions	*δ*c (CD_3_OD+ CDCl_3_, 100 MHz)	*δ*_H_ (mult; *J*) (CD_3_OD+ CDCl_3_, 400 MHz)	*δ*c (CD_3_Cl, 125 MHz)	*δ*_H_ (mult; *J*) (CD_3_Cl, 500 MHz)
1	32.9	1.40 (1H;*m*) 1.12 (1H; *m*)	38.5	1.64 (1H; *m*) 0.98 (1H; *m*)
2	24.5	1.99 (1H; *m*) 1.52 (1H; *m*)	27.1	1.62 (1H; *m*) 1.58 (1H; *m*)
3	75.4	3.35 (1H;*dd*; 11.9; 4.8)	78.9	3.22 (1H; dd; 11.5; 4.5)
4	37.1	–	38.7	–
5	48.8	1.30 (1H;*m*)	55.1	0.74 (1H; dd; 11.5; 1.5)
6	18.0	1.45 (1H; *m*) 1.44 (1H; *m*)	18.3	1.58 (1H; t; 3.6) 1.41 (1H; dd; 15.5; 12.0)
7	32.4	1.62 (1H; *m*) 1.38 (1H; *m*)	32.6	1.54 (1H; t; 3.5) 1.33 (1H; t; 3.6)
8	39.9	–	39.8	–
9	46.5	1.06 (1H; *m*)	46.8	1.51 (1H; dd; 11.0; 6.5)
10	36.6	–	37.3	–
11	23.4	1.91 (2H; *m*)	23.5	1.92 (1H; ddd; 18.5; 11.0; 3.5) 1.86 (1H; ddd; 18.5; 7.0; 4.0)
12	122.3	5.26 (1H; *t*; 3.4)	122.3	5.25 (1H; *t*; 3.5)
13	143.7	–	143.5	–
14	43.5	–	43.7	–
15	34.9	1.71 (1H; *m*) 1.26 (1H; *m*)	35.5	1.67 (1H; d; 13.0) 1.31 (1H; dd; 13.0; 5.0)
16	65.0	4.16 (1H; *dd*; 11.5; 4.9)	66.0	4.20 (1H; dd; 11.5; 5.0)
17	37.0	–	36.8	–
18	49.2	2.16 (1H; *dd*; 11.5; 4.9)	49.0	2.15 (1H; dd; 14.0; 4.5)
19	46.5	1.71 (1H; *m*) 1.06 (1H; *m*)	46.5	1.68 (1H; t; 14.0) 1.06 (1H; ddd; 13.5; 4.5; 2.5)
20	30.4	–	30.9	–
21	34.0	1.41 (1H; *m*) 1.13 (1H; *m*)	34.1	1.36 (1H; t; 3.7) 1.15 (1H; *t*; 3.6)
22	30.5	1.91 (1H; *m*) 1.88 (1H; *m*)	30.5	1.83(1H; t; 3.4) 1.20(1H; t; 3.5)
23	27.8	0.95 (3H; *s*)	28.0	1.00 (3H; *s*)
24	21.7	0.86 (3H; *s*)	15.6	0.79 (3H; *s*)
25	14.8	0.99 (3H; *s*)	15.5	0.94 (3H; *s*)
26	16.24	1.03 (3H; *s*)	16.8	0.99 (3H; *s*)
27	26.4	1.27 (3H; *s*)	27.1	1.22(3H; *s*)
28	21.4	0.80 (3H; *s*)	21.4	0.80 (3H; *s*)
29	32.6	0.90 (3H; *s*)	33.2	0.89 (3H; *s*)
30	23.2	0.92 (3H; *s*)	23.9	0.90 (3H; *s*)

**Table 3:** ^1^H-NMR and ^13^C-NMR of compound 2

Compound 2			Scopoletin, Mogana et al. [36]	
Positions	*δ*_C_ (acétone-*d*_6_, 100 MHz)	*δ*_H_ (mult; *J*) (acétone-*d*_6_, 400 MHz)	*δ*c (CD_3_Cl, 100 MHz)	*δ*_H_ (mult; *J*) (CD_3_Cl, 400 MHz)
1	–	–	–	–
2	160.4	–	161.6	–
3	112.5	6.20 (1H; *d*; 9.5)	111.6	6.30 (1H; *d*; 9.5)
4	143.6	7.86 (1H; *d*; 9.5)	143.3	7.63 (1H; *d*; 9.5)
5	102.8	6.81 (1H; *s*)	103.2	6.87 (1H; *s*)
6	144.9	–	144.6	–
7	150.8	–	150.2	–
8	108.9	7.20 (1H; *s*)	107.4	6.95 (1H; *s*)
9	150.0	–	149.7	–
10	112.1	–	113.5	–
6-OCH_3_	55.9	3.92 (3H; *s*)	56.4	3.98 (3H; *s*)
7-OH	–	8.78 (1H; *s*)	–	–

**Table 4:** ^1^H-NMR and ^13^C-NMR of compound 3

Compound 3			Ethyl gallate, Ooshiro et al. [37]	
Positions	*δ*c (CD_3_OD, 100 MHz)	*δ*_H_ (mult; *J*) (CD_3_OD, 400 MHz)	δc (CD_3_OD, 150 MHz)	*δ*_H_ (mult; *J*) (CD_3_OD, 600 MHz)
1	168.8	–	168.5	–
2	121.7	–	121.7	–
3/7	110.0	7.07 (2H; *s*)	110.0	7.04 (2H; *s*)
4/6	146.2	–	146.4	–
5	139.6	–	139.7	–
1’	61.6	4.28 (2H; *q*; 7.1)	61.6	4.28 (2H; *q*; 7.3)
2’	14.7	1.35 (3H; *t*; 7.1)	14.6	1.33 (3H; *t*; 7.3)

**Table 5:** ^1^H-NMR and ^13^C-NMR of compound 4

Compound 4			Gallic acid, Chanwitheesuk et al. [38]	
Positions	*δ*c (CD_3_OD, 100 MHz)	*δ*_H_ (mult; *J*) (CD_3_OD, 400 MHz)	*δ*c (acétone-*d*_6_, 100 MHz)	*δ*_H_ (mult; *J*) (acétone-*d*_6_, 400 MHz)
1	168.8	–	167.3	–
2	120.7	–	120.8	–
3/7	108.0	7.08 (2H; *s*)	109.1	7.15 (2H; *s*)
4/6	145.0	–	144.9	
5	138.1	–	137.7	

### Anti-salmonellal activity of partition fractions and isolated compounds from stem barks extract of *Canarium schweinfurthii*

Table 6 shows the inhibition parameters (MIC, MBC, MBC/MIC ratio) of the crude extract, partition fractions and isolated compounds of *Canarium schweinfurthii* against pathogenic *Salmonella*. The isolated compounds have variable activity (16 ≤ MIC≤1024 μg/mL) on the tested *Salmonella* serotypes. It appears that the activity of isolated compounds is greater than those of the crude extract and partitions. Among the partition fractions, chloroform and ethyl acetate fractions showed the best anti-salmonellal activity while among the isolated compounds, scopoletin showed the highest inhibitory activity with a MIC of 16 μg/mL against *Salmonella* Typhimurium and *Salmonella* Enteritidis. MIC values of other compounds and extract ranged between 128 and 1024 μg/mL, while hexane and residual fractions are the less active substances with MICs of 512 or 1024 μg/mL.

**Table 6:** Inhibition parameters (MIC, MBC) of partition fractions and isolated compounds from *Canarium schweinfurthii* against different test microorganisms

Tested samples	Studied parameters (μg/mL)	Strain/isolates			
		ST	STs	STM	SE
**HEE 50/50**	MIC	256	128	64	128
	MBC	512	512	256	512
	MBC/MIC	2	4	4	4
**Hexane partition**	MIC	1024	1024	512	> 1024
	MBC	> 1024	> 1024	> 1024	> 1024
	MBC/MIC	–	–	–	–
**Chloroform partition**	MIC	512	1024	256	1024
	MBC	1024	> 1024	> 1024	> 1024
	MBC/MIC	2	–	–	–
**Ethyle acetate partition**	MIC	256	256	128	32
	MBC	> 1024	1024	> 1024	128
	MBC/MIC	–	4	–	4
**n-butanol partition**	MIC	> 1024	1024	512	> 1024
	MBC	> 1024	> 1024	> 1024	> 1024
	MBC/MIC	–	–	–	–
**Residual partition**	MIC	> 1024	> 1024	> 1024	1024
	MBC	> 1024	512	256	> 1024
	MBC/MIC	–	–	–	–
**Compound 1** **Maniladiol**	MIC	512	512	32	64
	MBC	> 1024	> 1024	128	256
	MBC/MIC	–	–	4	4
**Compound 2** **Scopoletin**	MIC	32	32	16	16
	MBC	64	128	32	64
	MBC/MIC	2	4	2	4
**Compound 3** **Ethyl gallate**	MIC	128	1024	64	1024
	MBC	> 1024	> 1024	> 1024	> 1024
	MBC/MIC	–	–	–	–
**Compound 4** **Gallic acid**	MIC	32	32	64	128
	MBC	32	32	128	256
	MBC/MIC	1	1	2	2
**Oxytetracycline**	MIC	8	8	4	2
	MBC	32	64	32	16
	MBC/MIC	4	8	8	8
**Ciprofloxacine**	MIC	0,5	1	4	4
	MBC	2	2	8	8
	MBC/MIC	4	2	2	2

*ST Salmonella* Typhi, *STs Salmonella* Typhi ATCC6539, *STM Salmonella* Typhimurium, *SE Salmonella* Enteritidis, *MIC* Minimum inhibitory concentration, *MBC* Minimum bactericidal concentration.

## Discussion

The antimicrobial effects of some plants and their extracts are well known today [39, 40]; the diversity of plant species is a valuable source for the search for new classes of antibiotics. These plants may proffer valuable alternative to address certain human and veterinary health challenges. It is in this perspective that the hydro-ethanolic extract of *Canarium schweinfurthii* has been explored for its anti-salmonellal activity and its bioactive compounds. Several plants are traditionally used against human salmonellosis [41–46] and avian salmonellosis [24–26, 47]. Plants with high anti-salmonellal potential that show promise for the control of avian salmonellosis include *Aloe secundiflora* [47], *Thymus vulgaris* [48], *Curcuma longa* and *Scutellaria baicalensis* [25] and *Erica mannii* [27]. Plant extracts as well as traditionally improved drugs are one of the promising ways to combat human salmonellosis [23, 47, 49]. Several authors [22, 23, 28, 50–53] have shown that plant extracts depending on their concentrations are active both in vitro and in vivo against several *Salmonella* serotypes. Most of these extracts treat salmonellosis in the same range of time as conventional medicines. These findings corroborate our results which showed that the hydroethanolic extract of *Canarium schweinfurthii* is active against *Salmonella* serotypes with MIC range from 64 to 128 μg/ml, moreover this extract have previously demonstrated an in vivo anti-salmonellal activity [20], curing avian salmonellosis on day 9 and with the doses 19 and 75 mg/kg bw of the extract. In addition to the therapeutic efficacy of the hydroethanolic extract of *Canarium schweinfurthii*, the antibacterial activity of its partitions was evaluated. Among the partitions, chloroform and ethyl acetate fractions showed the best anti-salmonellal activity. It also appears that the activity of isolated compounds is greater than those of the crude extract and partitions. This could be due to the low concentration of these compounds in the plant extract or to the antagonism effect of other compounds present in the same extract. The anti-salmonellal activity of plants is linked to the diversity and complexity of their secondary metabolites. The in vitro anti-salmonellal effect of hydroethanolic extract of *Canarium schweinfurthii* found in this study and its therapeutic efficacy [20] can be linked to a combined action of its secondary metabolites. Indeed, at the molecular level, compounds such as gallic acid and scopoletin found in plants belonging to *Canarium* genus [54] could act synergistically and could be partly responsible for the anti-infectious activity of *Canarium schweinfurthii*. In order to verify this possibility and to have a clear idea on the active principles of this plant, the fractionation of its stem bark extract was performed.

Gallic acid, ethyl gallate, scopoletin and maniladiol were isolated from the *Canarium schweinfurthii* stem bark extract, these compounds were reported for the first time in this medicinal plant species and belong to the classes of polyphenols, triperpenes and coumarins. From the previous reports [54], only gallic acid and scopoletin have been isolated from other plants belonging to the same genus as *Canarium schweinfurthii* and these compounds were reported to have antibacterial and antioxidant properties. The isolated compounds have variable activities (16 ≤ MIC≤1024 μg/mL) against the tested *Salmonella* serotypes. Among the pure isolated compounds, scopoletin showed the highest inhibitory activity with a MIC of 16 μg/mL against *Salmonella* Typhimurium and *Salmonella* Enteritidis. The activity of most of the isolated compounds was less than those of oxyphylline B (10 μg/mL) isolated from *Zizyphus oxyphylla* Edgew against *Salmonella* Typhi [55] and lespedin **(**12.25 μg/ml) isolated from *Brillanta isialamium* against *Salmonella* Typhi [56]. However the anti-salmonellal activity of gallic acid and scopoletin against *Salmonella* Typhi (32 μg/mL) was better than those of Bafoudiosbulbins A and Bafoudiosbulbins B isolated from *Dioscorea bulbifera* L. var. sativa [57]. These results corroborate the finding of Lunga et al. [44] who showed that the anti-salmonellal activity of isolated compounds from *Paullinia pinnata* Linn ranged from 0.781 to 100 μg/mL. According to the Kuete’s classification scale [39], the antibacterial activity of a compound is significant when the MIC< 10 μg/mL; moderate when 10 < MIC≤100 μg/mL and low when MIC> 100 μg/ml. With regard to this scale, the anti-salmonellal activities of the isolated compound from *Canarium schweinfurthii* are moderate (10 < MIC≤100 μg/mL). Scopoletin and gallic acid are significantly active against *Salmonella* Typhi, *Salmonella* Typhi ATCC6539 and *Salmonella* Typhimurium. These results corroborate those of Okoli et al. [58] who showed that 3β-hydroxylolean-12,18-diene isolated from *Canarium schweinfurthii* was active on *Salmonella* with a MIC of 12.5 μg/ml against *Salmonella* Typhi. It has been shown that in addition to its immunomodulatory effect [59], scopoletin reduces the intracellular survival of *Salmonella* Typhi within U937 human macrophage cell line [60]. Gallic acid has in addition to its in vitro and in vivo antibacterial effect against *Salmonella* Typhimurium [61, 62], an antioxidant activity. These compounds related properties corroborate the findings of Sokoudjou et al. [20] who reported that the ability of the extract of *Canarium schweinfurthii* to cure salmonellosis in broilers could be explained by its ability to directly kill *Salmonella* and/or boost the immune system of the host. The dosage of the compounds isolated from this plant can be used to normalize the extract during the phytomedicine evaluation and preparation.

## Conclusion

Gallic acid, ethyl gallate, scopoletin and maniladiol were isolated from the *Canarium schweinfurthii* stem bark extract. These compounds were reported for the first time in this plant species. The four isolated compounds showed in vitro anti-salmonellal activity against *Salmonella* serotypes and particularly scopoletin was the most active and highly selective against both non-typhoidal *Salmonella* and typhoidal *Salmonella* with MIC of 16 or 32 μg/mL. The anti-salmonellal activity of the compounds isolated from *Canarium schweinfurthii* justifies the use of this plant in traditional medicine and confirms the anti-salmonellal effect of the hydroethanolic extract thus adding credence to its use in the treatment of avian salmonellosis. Further studies will be necessary to verify the in vivo activity of these compounds and to elucidate their mechanisms of action.

## Supplementary information

**Additional file 1.**

## Data Availability

They are available as Supporting information.
