# Supplementary material for: Isolation, characterization and in vitro anti-salmonellal activity of compounds from stem bark extract of Canarium schweinfurthii
Source: BMC Complement Med Ther. 2020 Oct 19;20:316. doi: 10.1186/s12906-020-03100-5 (PMC7574196; doi:10.1186/s12906-020-03100-5)
Supplement: Supplementary file 1 — Additional file 1. [file 12906_2020_3100_MOESM1_ESM.doc]

**Supporting Information**

Isolation, characterization and in vitro anti-salmonellal activity of compounds from stem bark extract of Canarium schweinfurthii

Jean Baptiste SOKOUDJOU^1,2^, Olubunmi ATOLANI^2,3^, Guy Sedar Singor NJATENG^1^, Afsar KHAN^2^, Cyrille NGOUFACK TAGOUSOP^4^, André Nehemie BITOMBO^2,5^, Norbert KODJIO^1^, Donatien GATSING^1*^

^1^Microbiology and Antimicrobial Substances Research Unit, Faculty of Science, University of Dschang, P.O. Box 67: Dschang, Cameroon.

^2^Natural Product Chemistry Laboratory, Department of Chemistry, COMSATS University Islamabad, Abbottabad Campus-22060, Pakistan.

^3^Department of Chemistry, Faculty of Physical Sciences, University of Ilorin, P.M.B. 1515, Ilorin, Nigeria

^4^Department of Basic Scientific Studies, University Institute of Technology, University of Ngaoundere, P.O. Box 455, Ngaoundere, Cameroon 

^5^Department of Organic Chemistry, Faculty of Science, University of Yaoundé I, P.O. Box 812, Yaoundé, Cameroon.

^*^*Corresponding author:* Donatien GATSING, *E-mail: gatsingd@yahoo.com, Tel: +237 677 51 67 40,* P.O. Box 67 Dschang, Cameroon.

*Corresponding author: Donatien GATSING, E-mail: gatsingd@yahoo.com, Tel: +237 677 51 67 40, P.O. Box 67 Dschang, Cameroon

| **Content** | **Page** |
| --- | --- |
| Figure 1. ^1^H NMR spectrum of compound **1** | 3 |
| Figure 2. ^13^C NMR spectrum of compound **1** | 3 |
| Figure 3. DEPT 90 spectrum of compound **1** | 4 |
| Figure 4. DEPT 135 spectrum of compound **1** | 4 |
| Figure 5. HSQC spectrum of compound **1** | 5 |
| Figure 6. HMBC spectrum of compound **1** | 5 |
| Figure 7. ^1^H-^1^H COSY spectrum of compound **1** | 6 |
| Figure 8. ROESY spectrum of compound **1** | 6 |
| Figure 9. ESIMS of compound **1** | 7 |
| Figure 10. ^1^H NMR spectrum of compound **2** | 7 |
| Figure 11. ^13^C NMR spectrum of compound **2** | 8 |
| Figure 12. DEPT 90 spectrum of compound **2** | 8 |
| Figure 13. DEPT 135 spectrum of compound **2** | 9 |
| Figure 14. HSQC spectrum of compound **2** | 9 |
| Figure 15. HMBC spectrum of compound **2** | 10 |
| Figure 16. ^1^H-^1^H COSY spectrum of compound **2** | 10 |
| Figure 17. ROESY spectrum of compound **2** | 11 |
| Figure 18. ESIMS of compound **2** | 11 |
| Figure 19. ^1^H NMR spectrum of compound **3** | 12 |
| Figure 20. ^13^C NMR spectrum of compound **3** | 12 |
| Figure 21. DEPT 135 spectrum of compound **3** | 13 |
| Figure 22. HSQC spectrum of compound **3** | 13 |
| Figure 23. ESIMS of compound **3** | 14 |
| Figure 24. ^1^H NMR spectrum of compound **4** | 14 |
| Figure 25. ^13^C NMR spectrum of compound **4** | 15 |
| Figure 26. ESIMS of compound **4** | 15 |


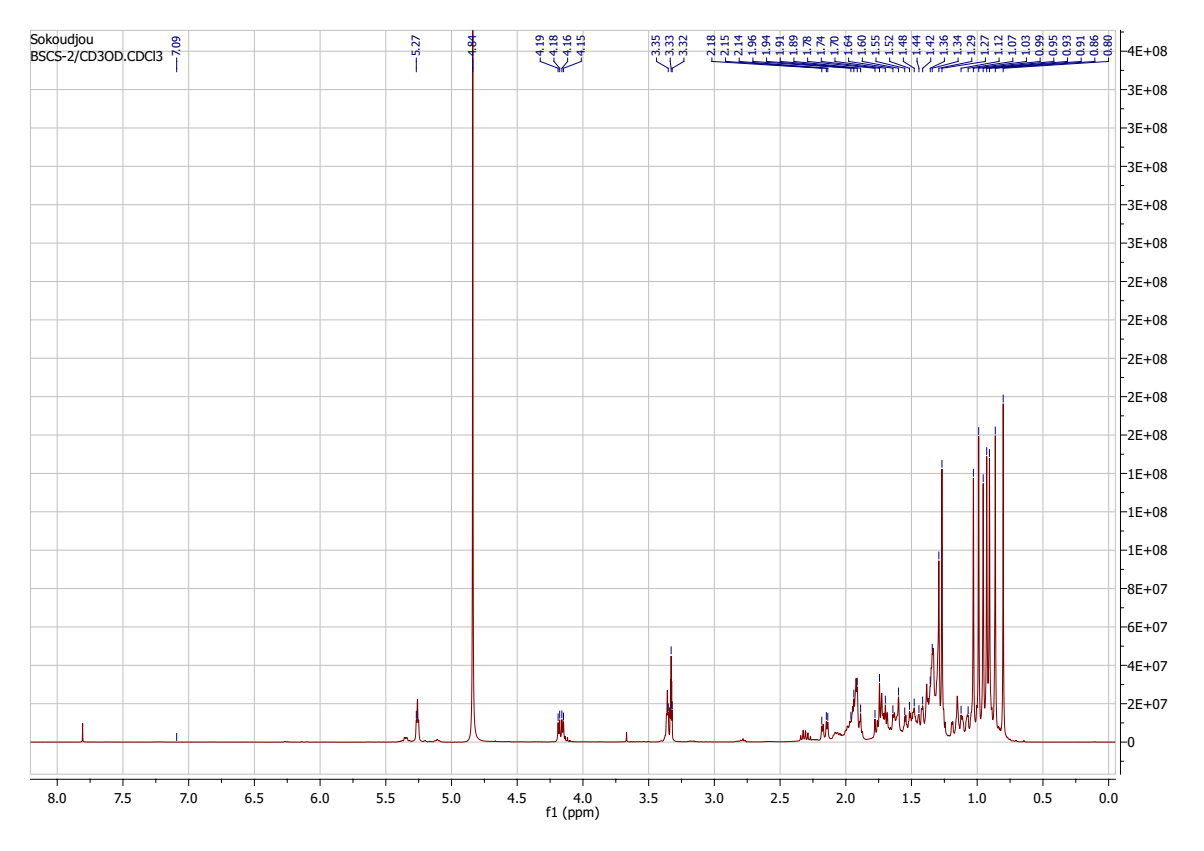


Figure 1. ^1^H NMR spectrum of compound **1**


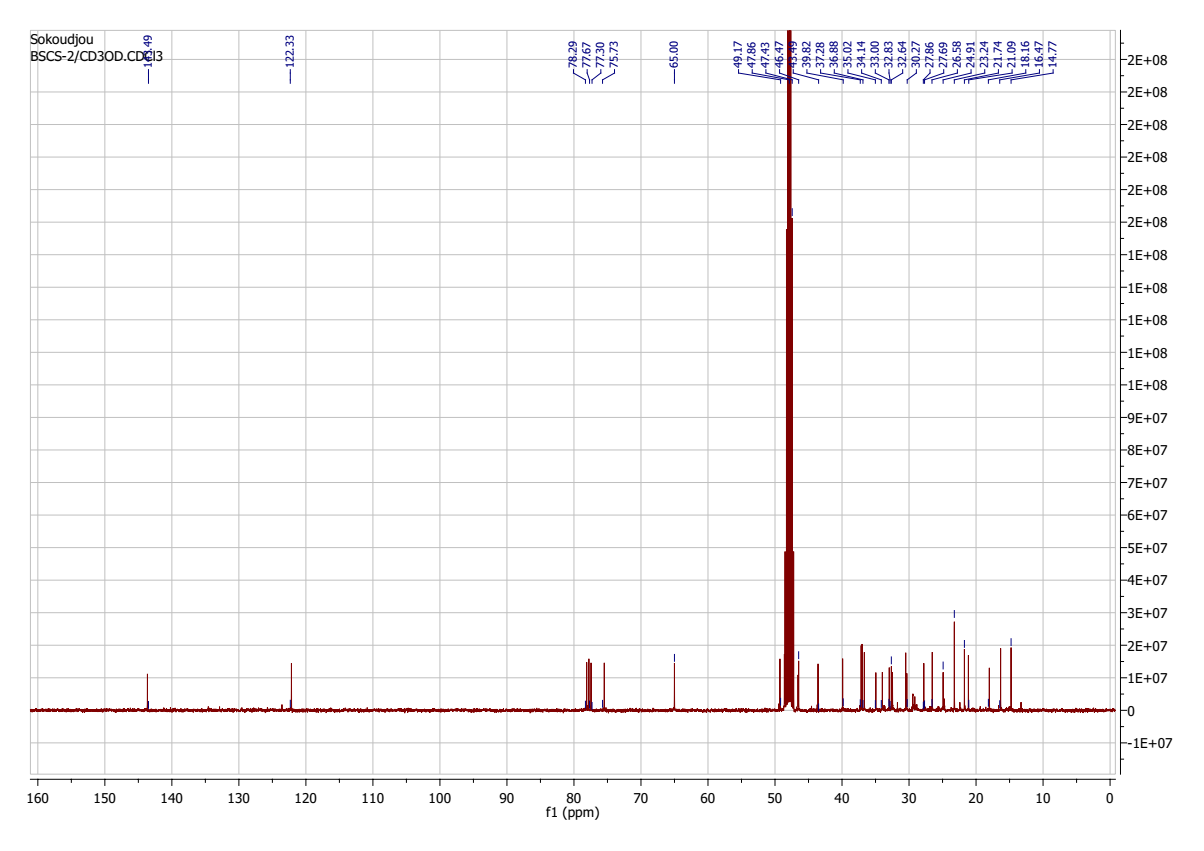
 Figure 2. ^13^C NMR spectrum of compound **1**


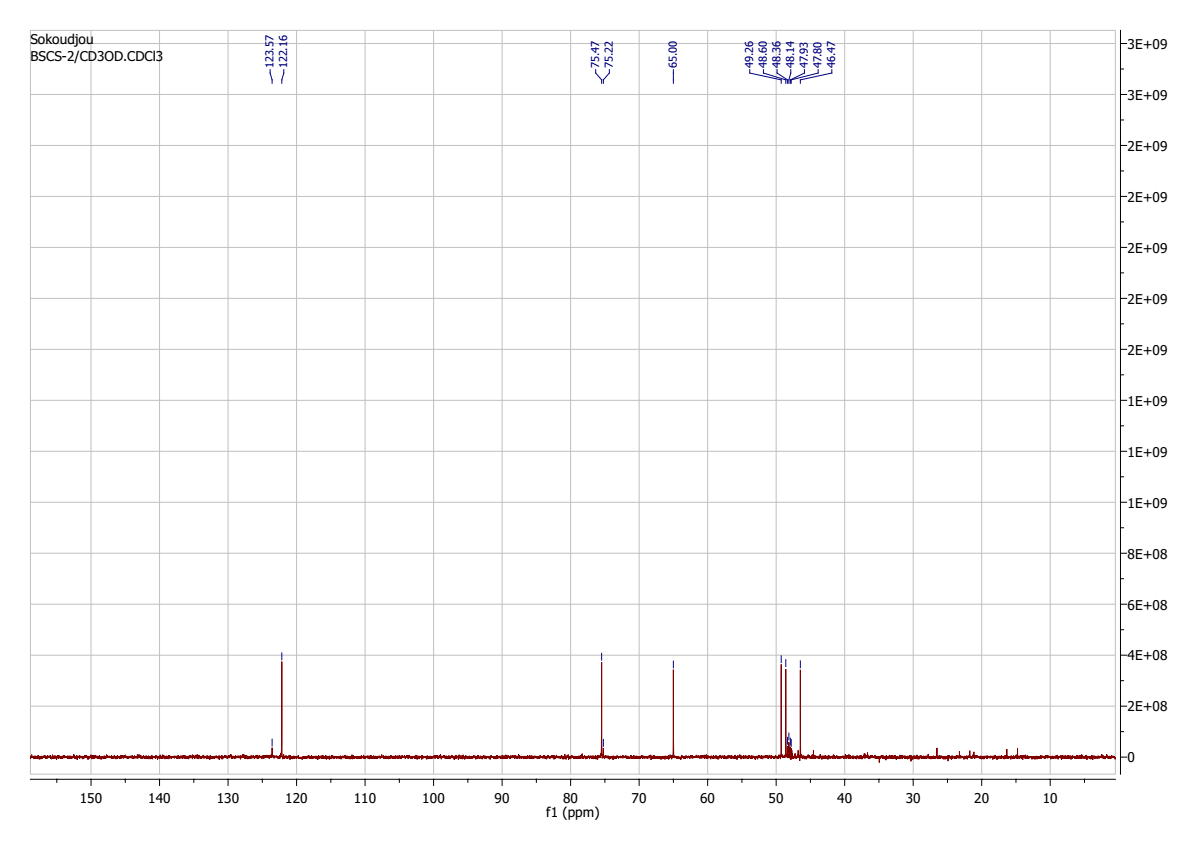
 Figure 3. DEPT 90 spectrum of compound **1**


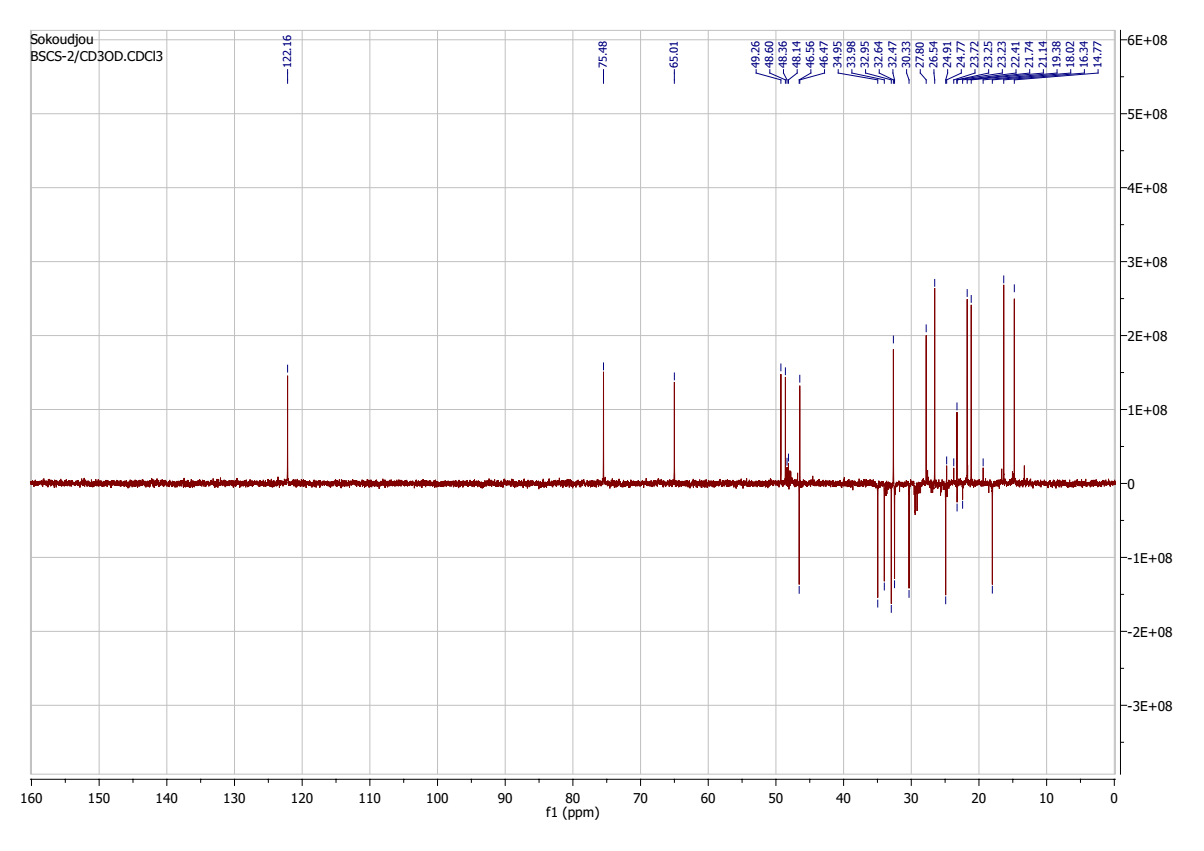
 Figure 4. DEPT 135 spectrum of compound **1**


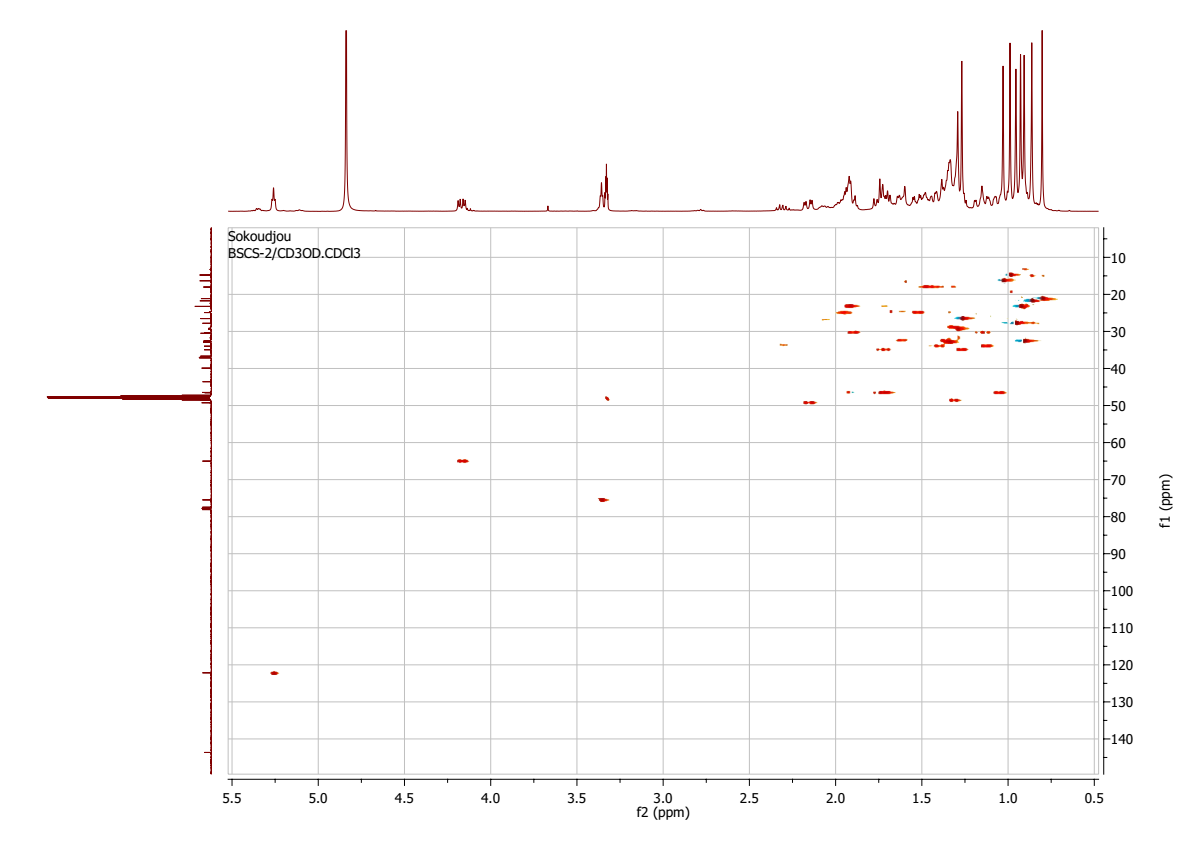
 Figure 5. HSQC spectrum of compound **1**


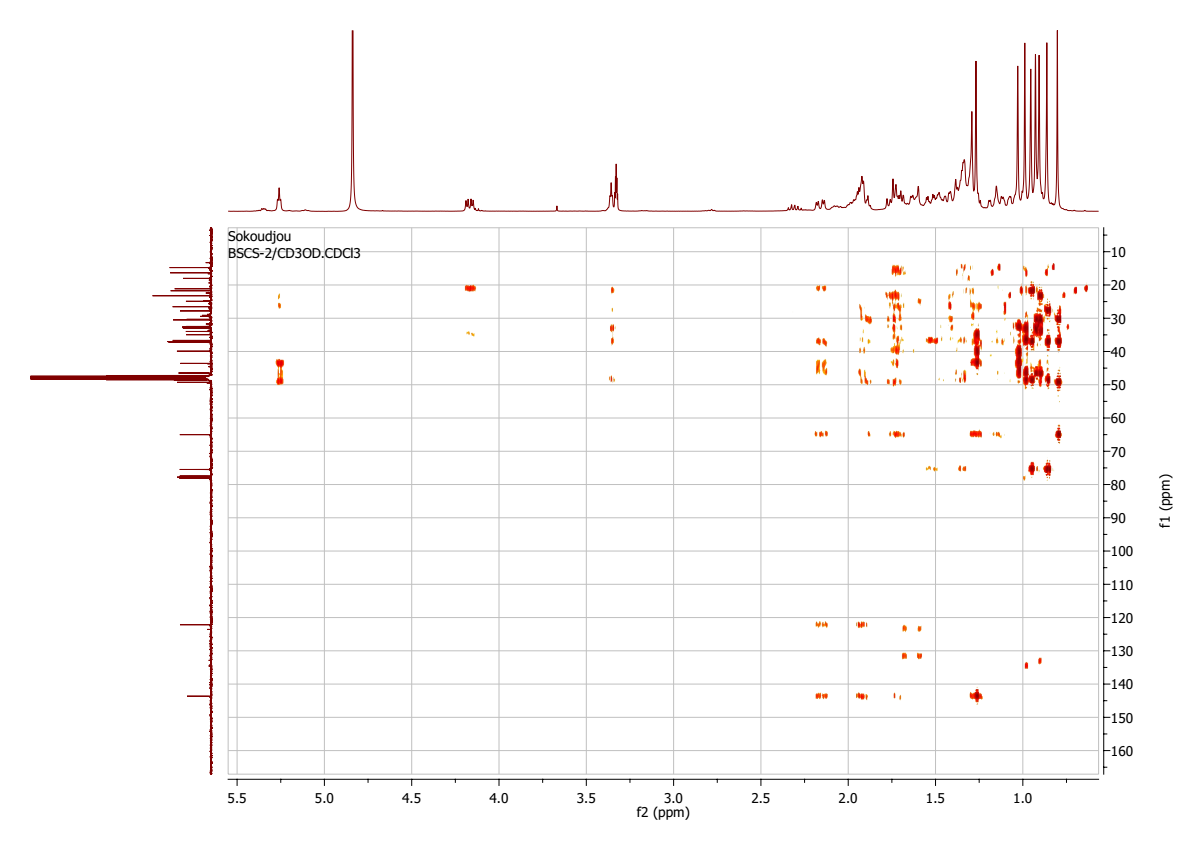
 Figure 6. HMBC spectrum of compound **1**


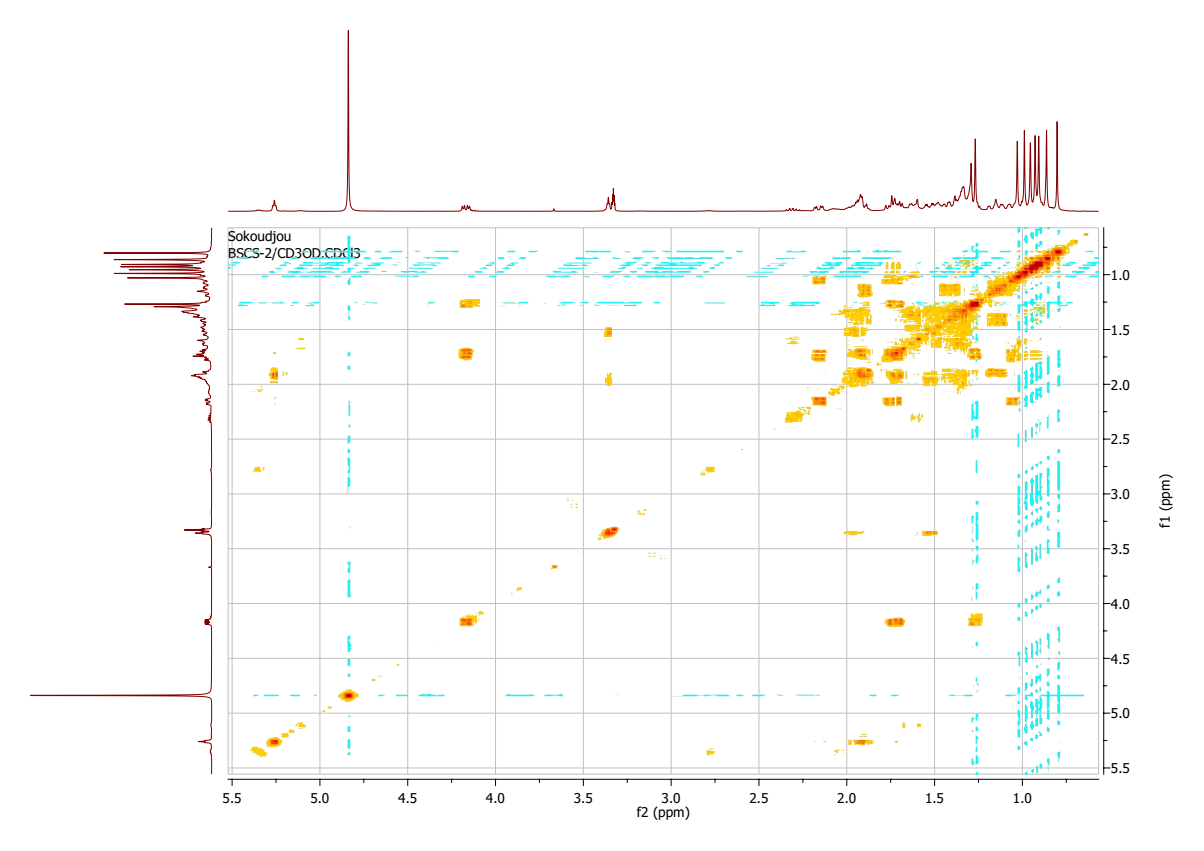
 Figure 7. ^1^H-^1^H COSY spectrum of compound **1**


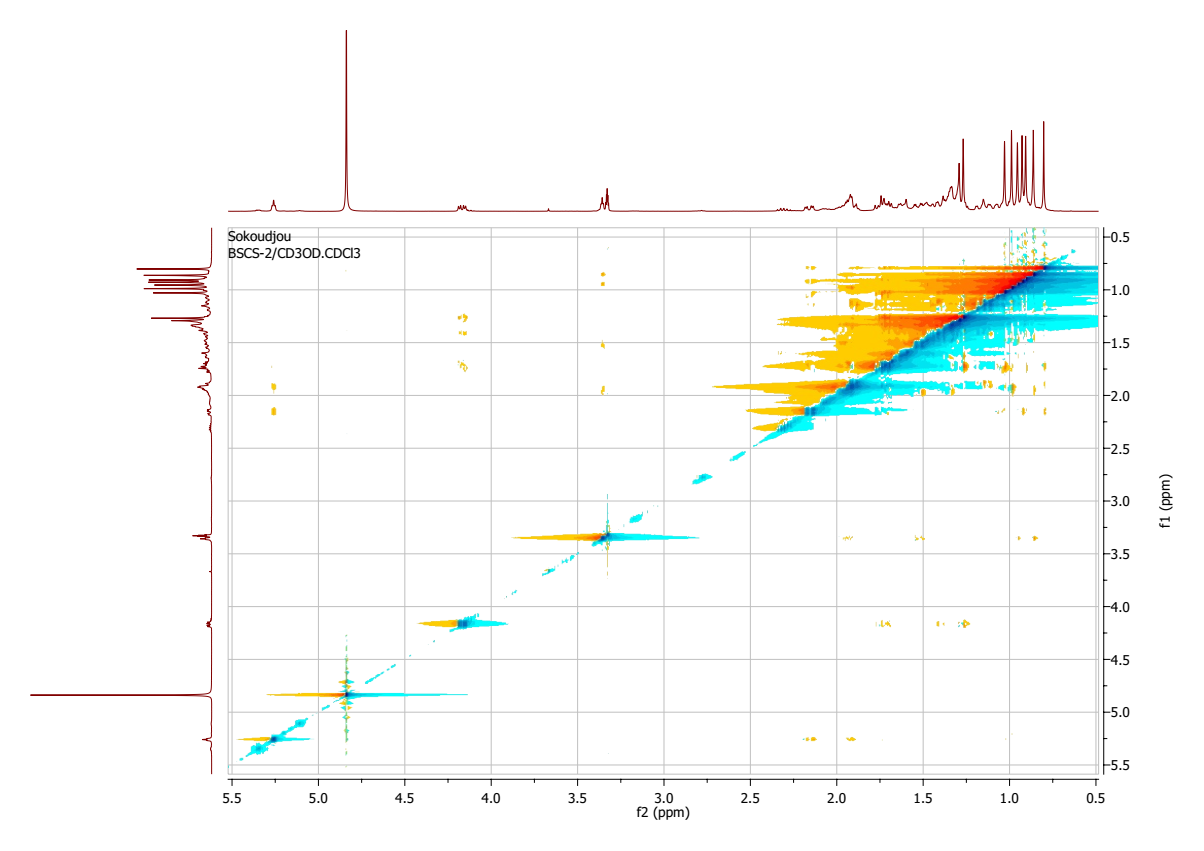
 Figure 8. ROESY spectrum of compound **1**


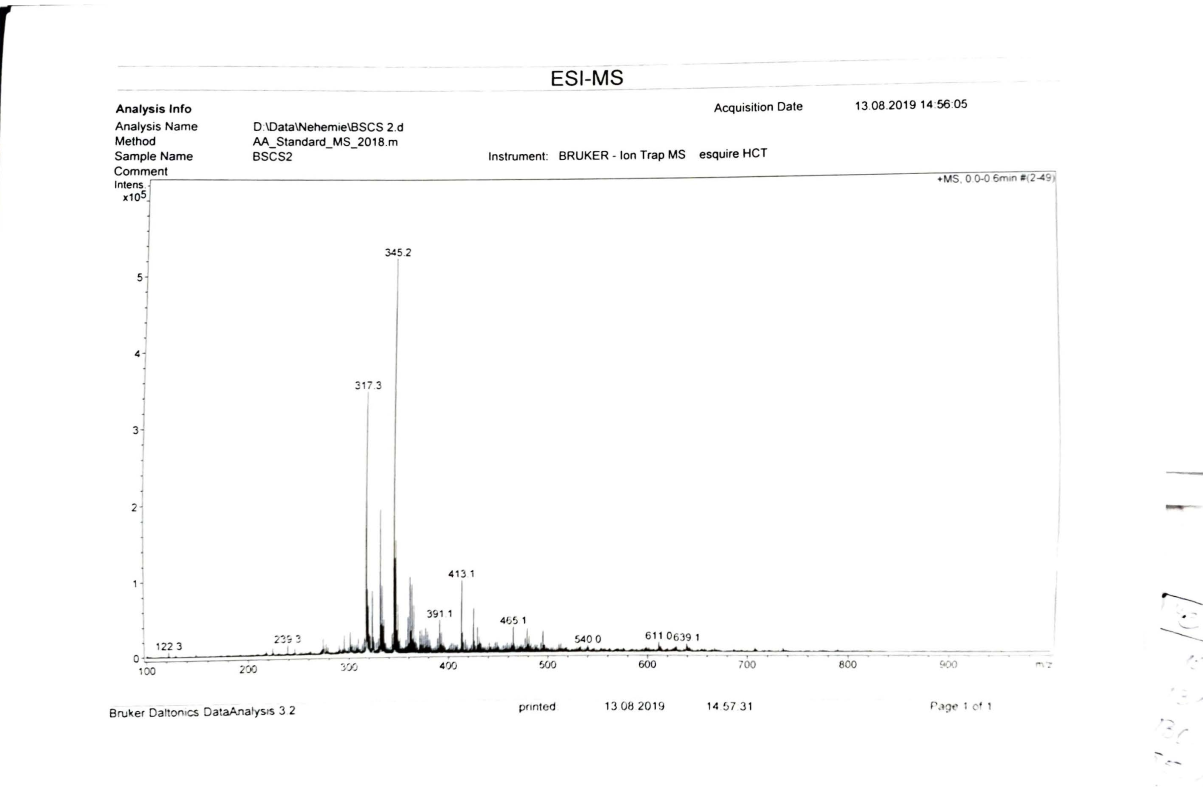
 Figure 9. ESIMS of compound **1**


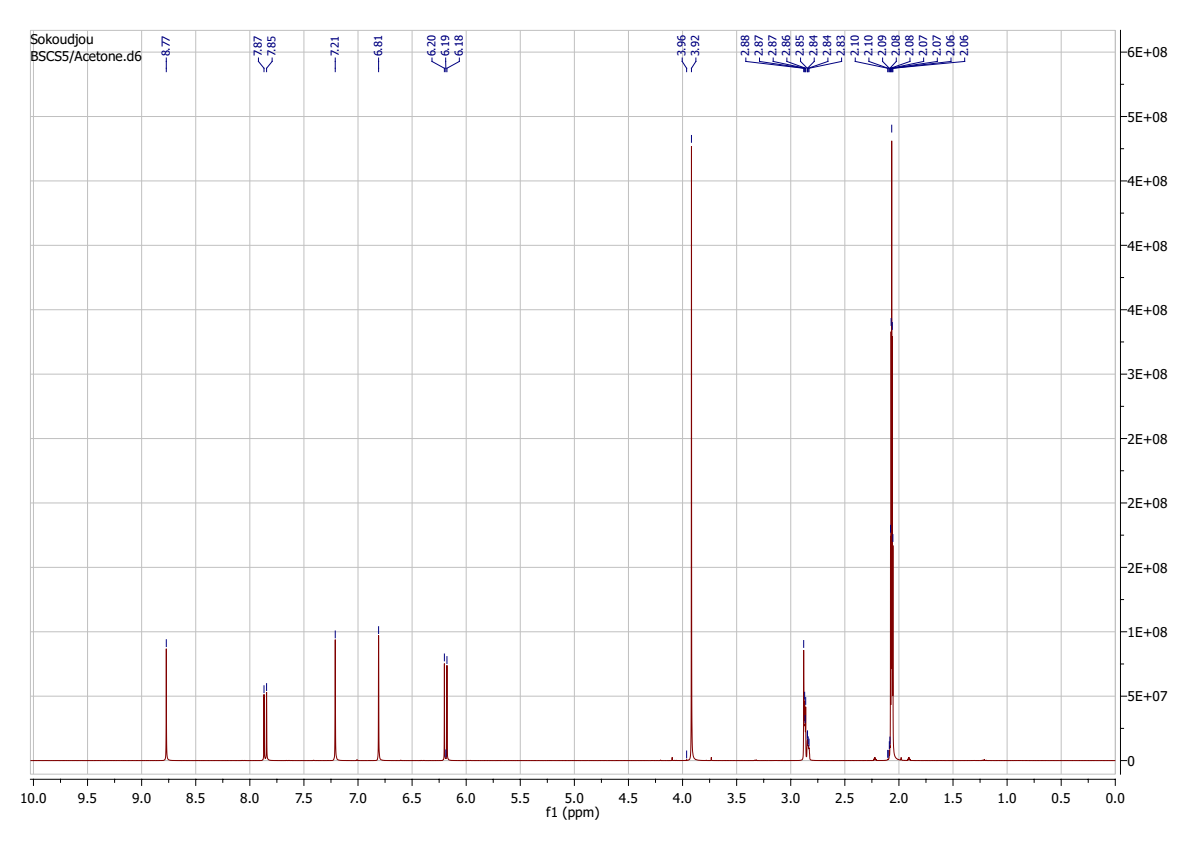


Figure 10. ^1^H NMR spectrum of compound **2**


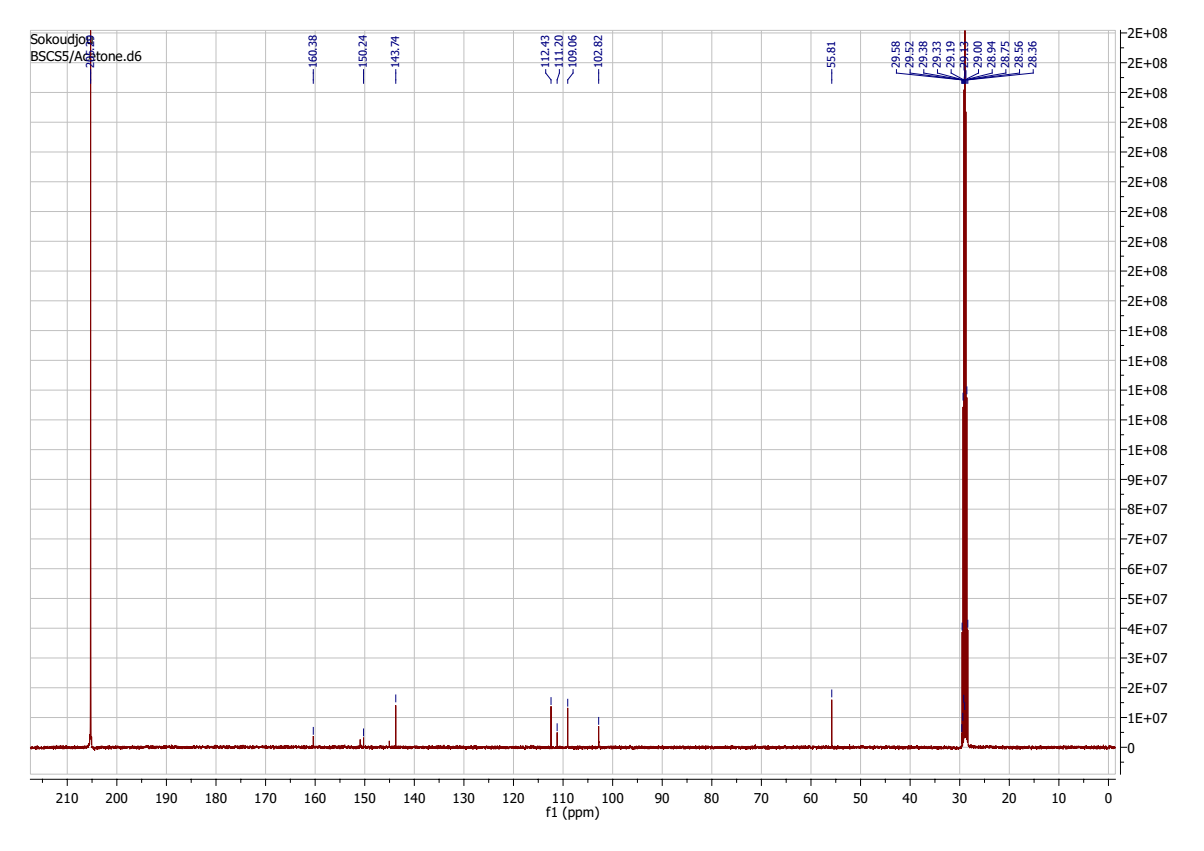
 Figure 11. ^13^C NMR spectrum of compound **2**
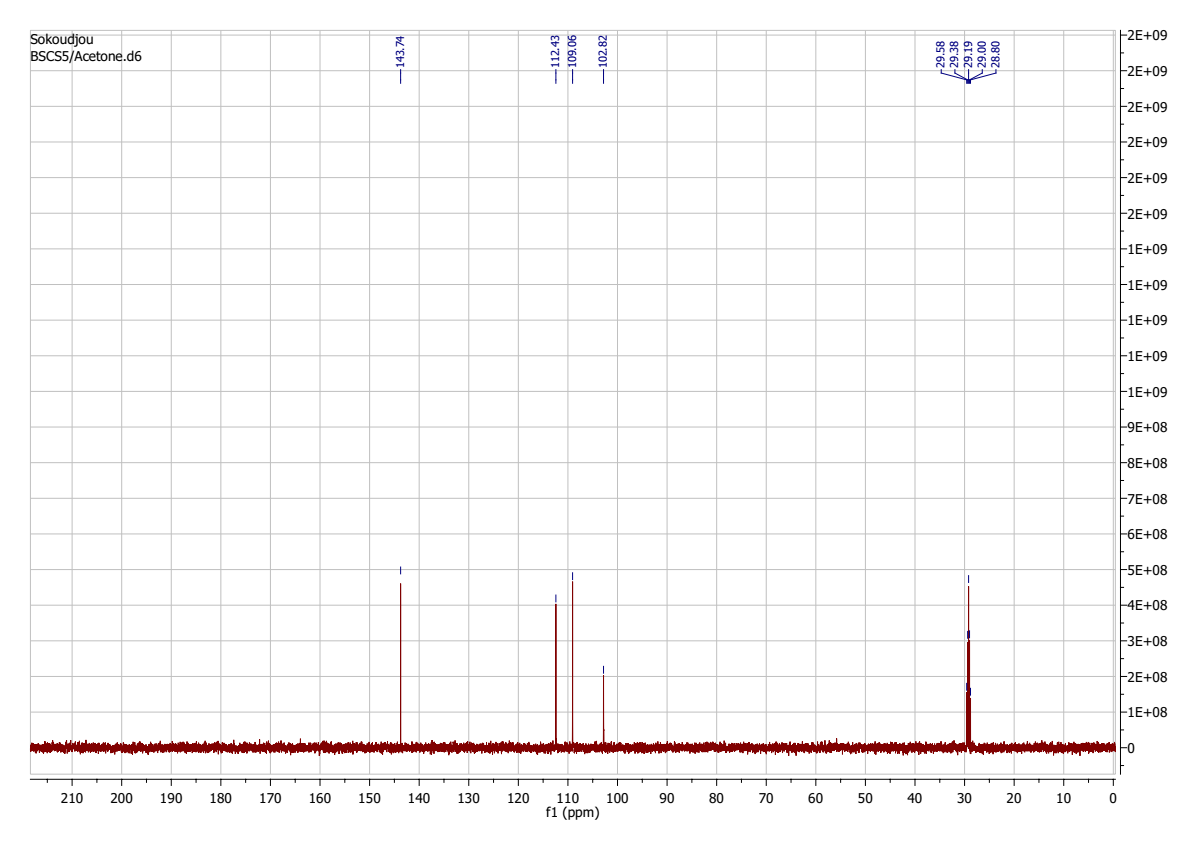


Figure 12. DEPT 90 spectrum of compound **2**


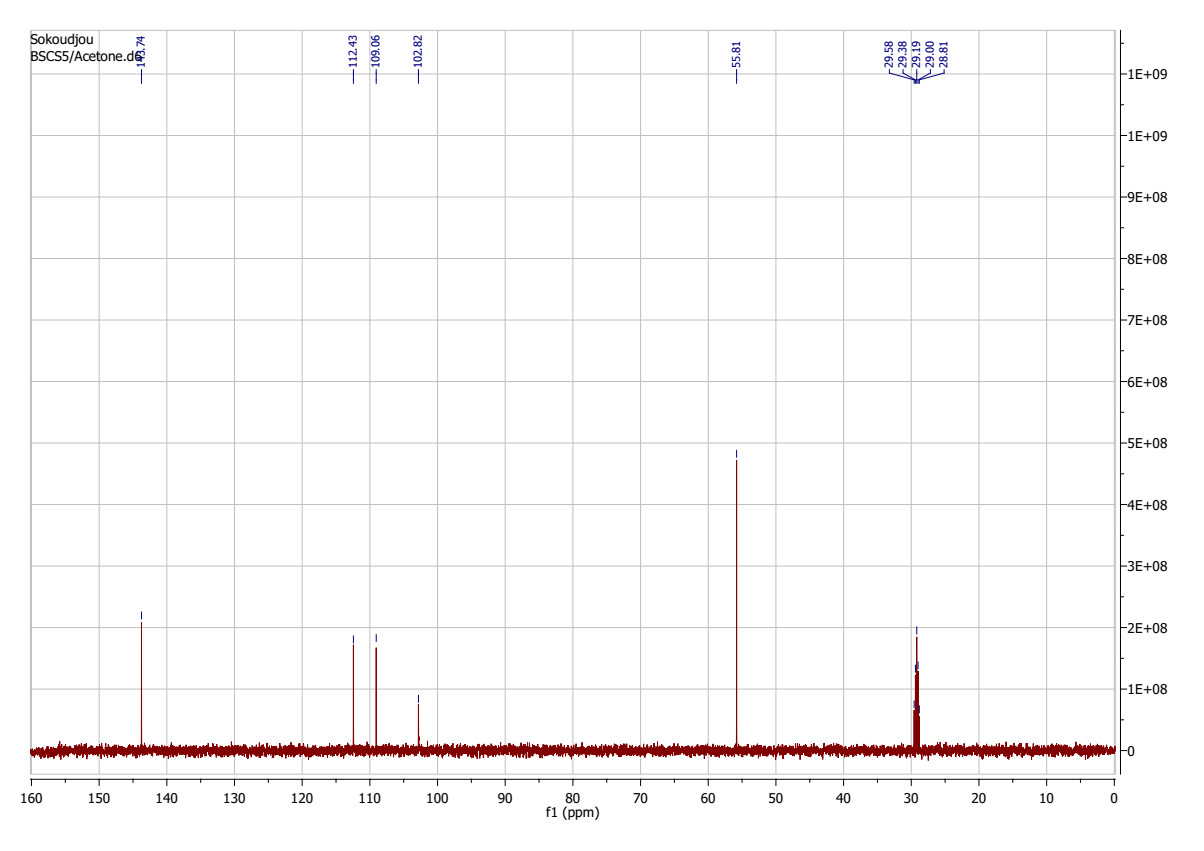
 Figure 13. DEPT 135 spectrum of compound **2**


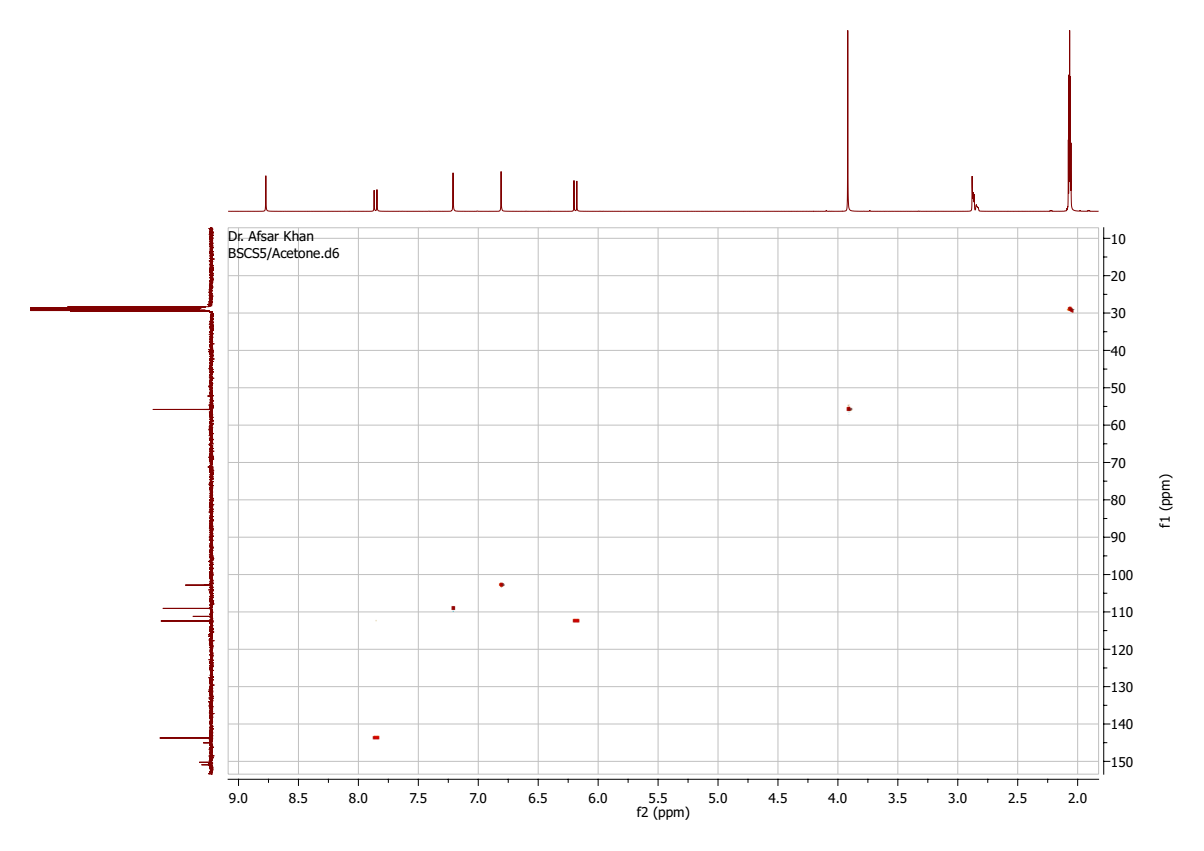
 Figure 14. HSQC spectrum of compound **2**

Figure 15. HMBC spectrum of compound **2**
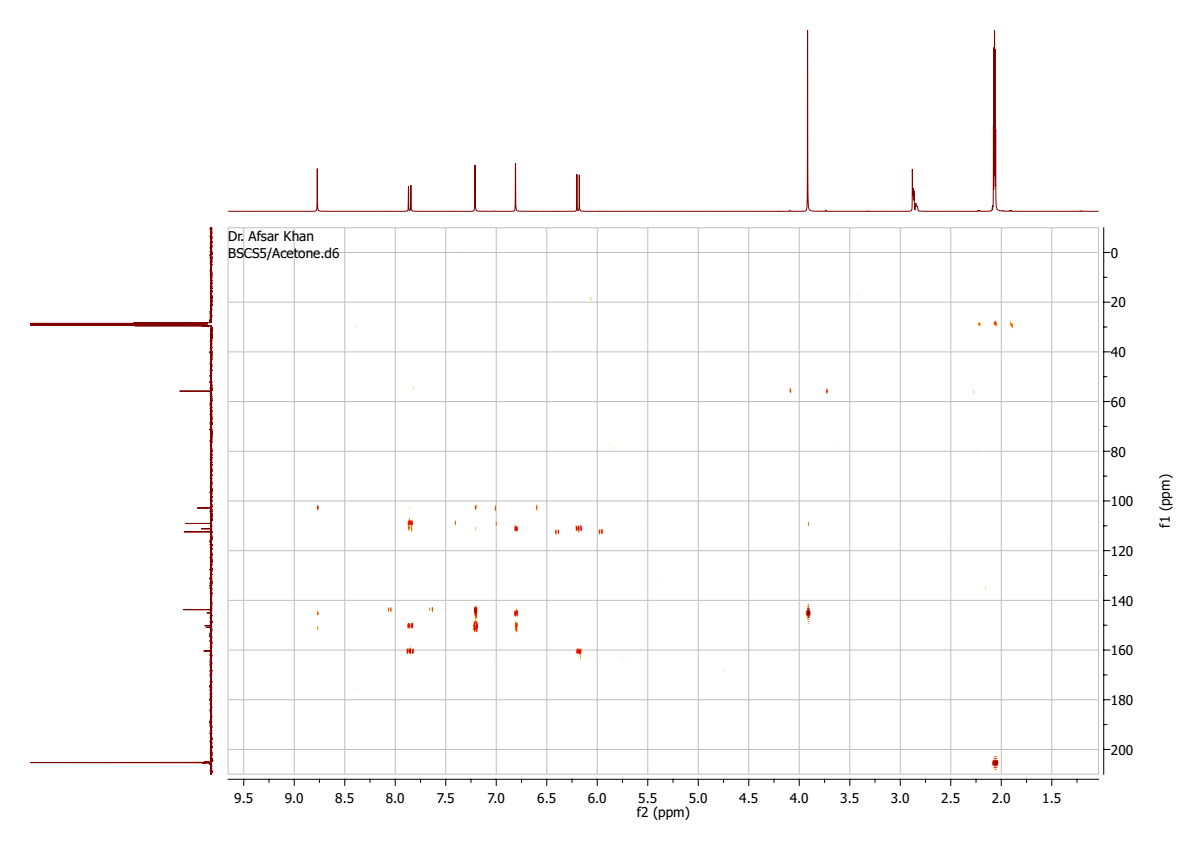


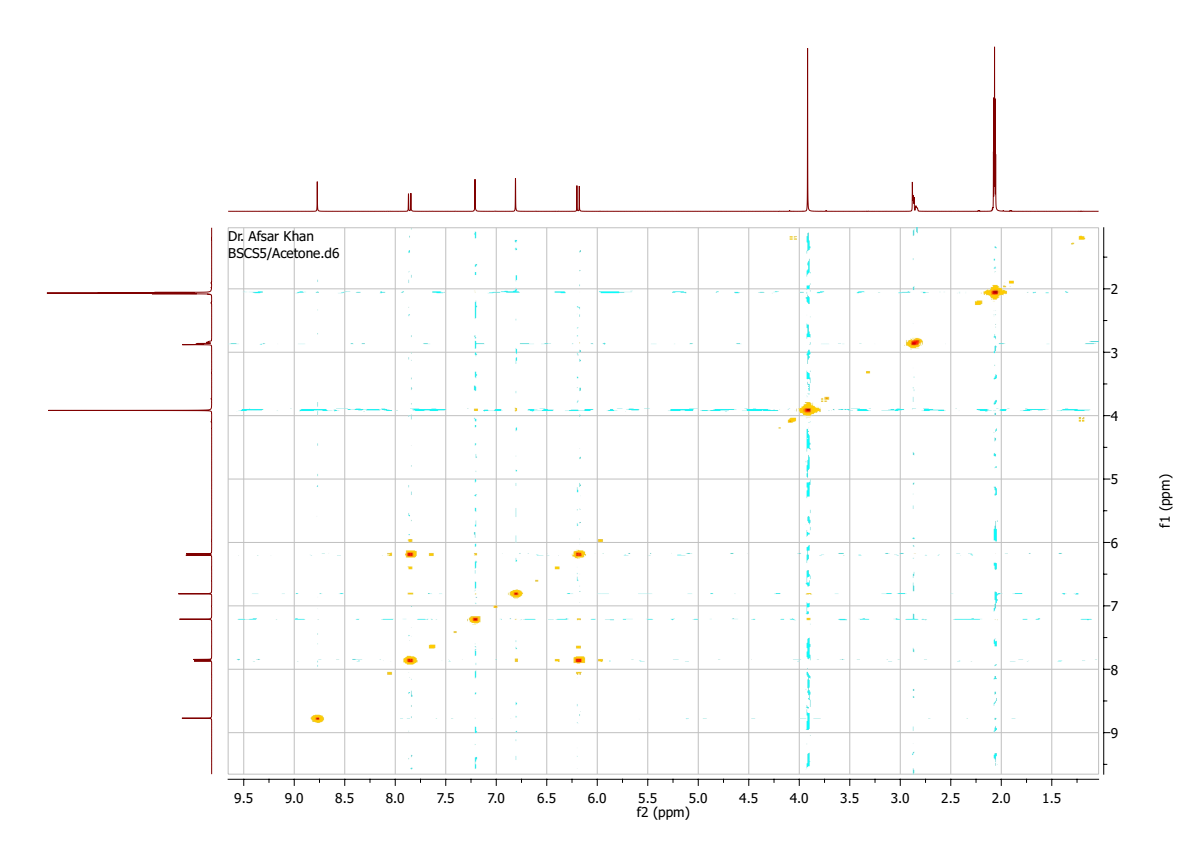
 Figure 16. ^1^H-^1^H COSY spectrum of compound **2**


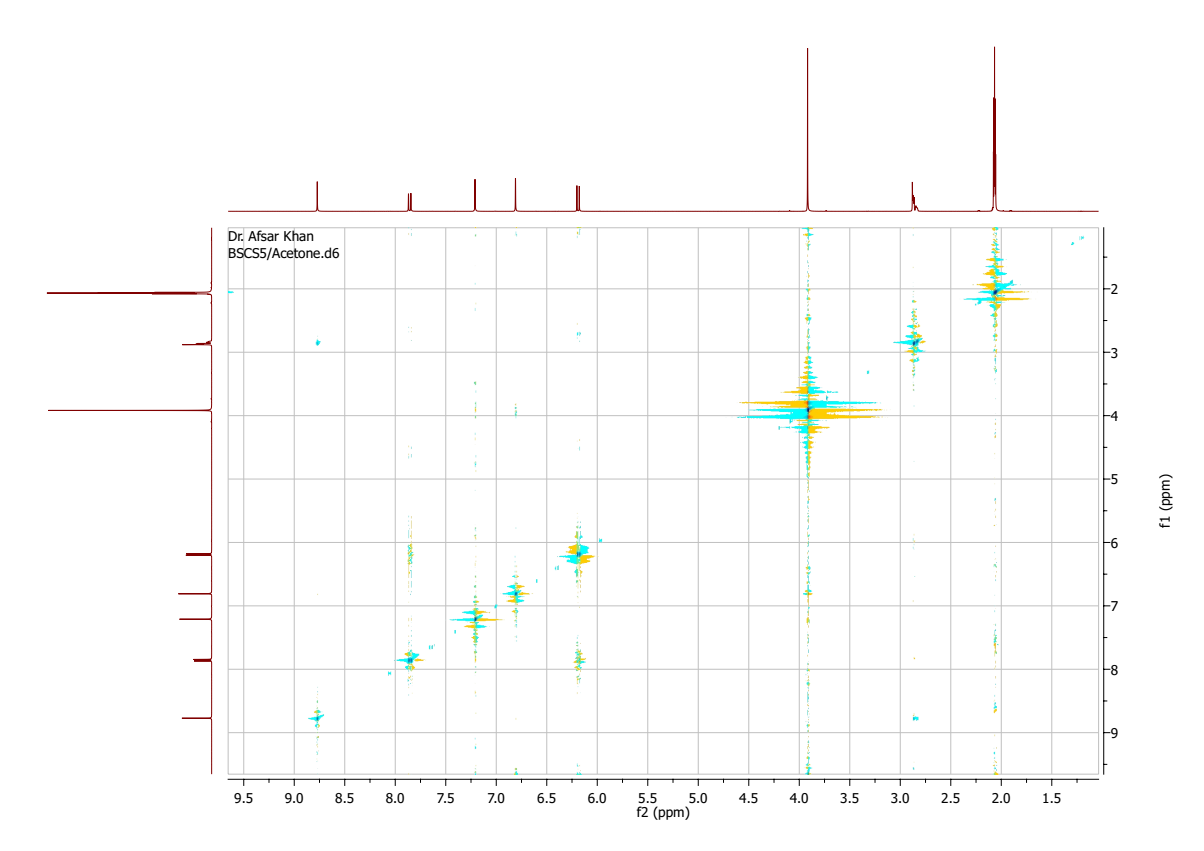
 Figure 17. ROESY spectrum of compound **2**


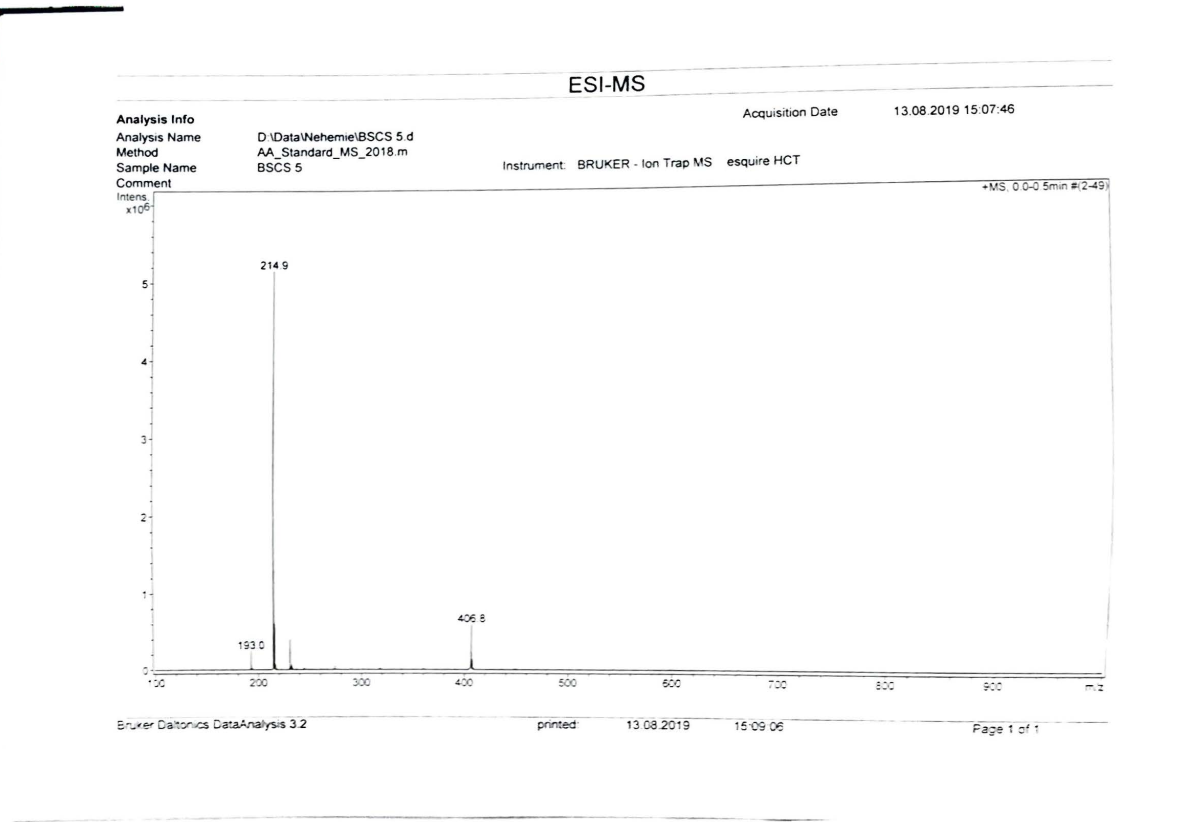


Figure 18. ESIMS of compound **2**


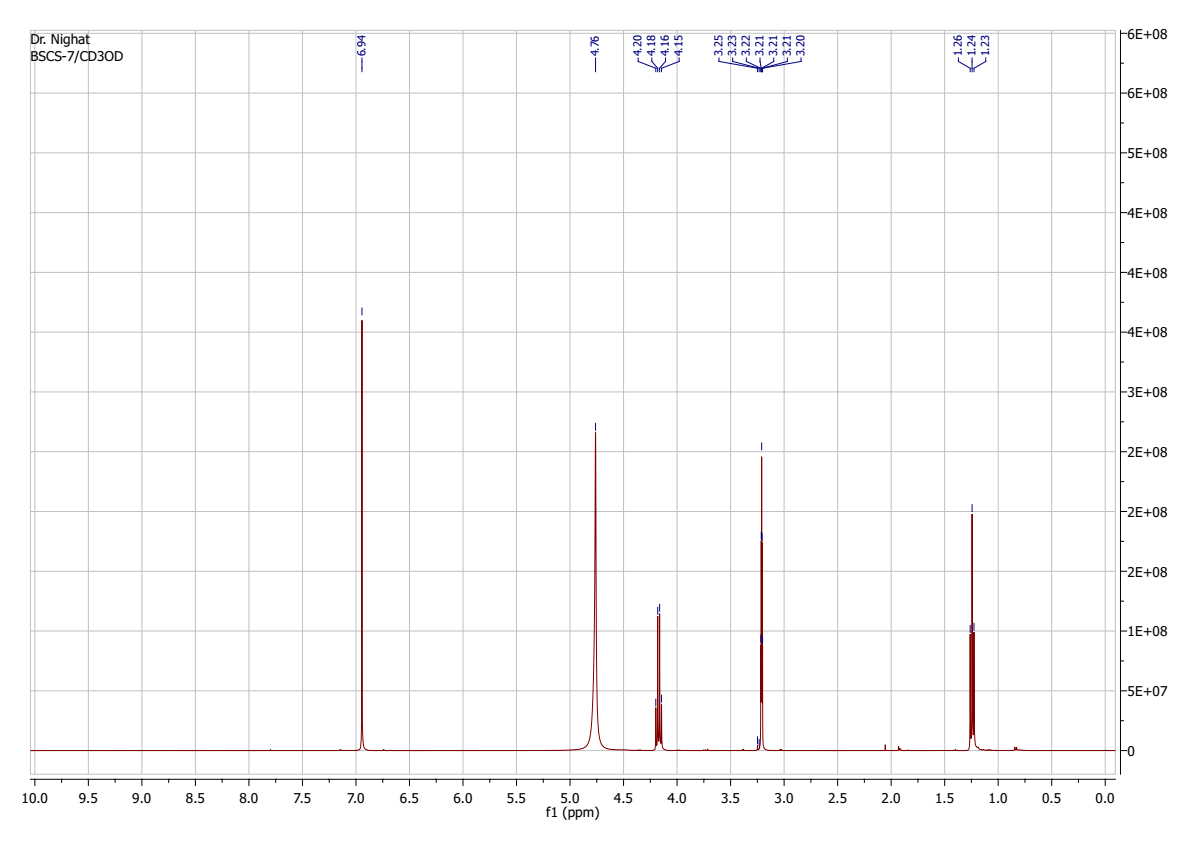
 Figure 19. ^1^H NMR spectrum of compound **3**


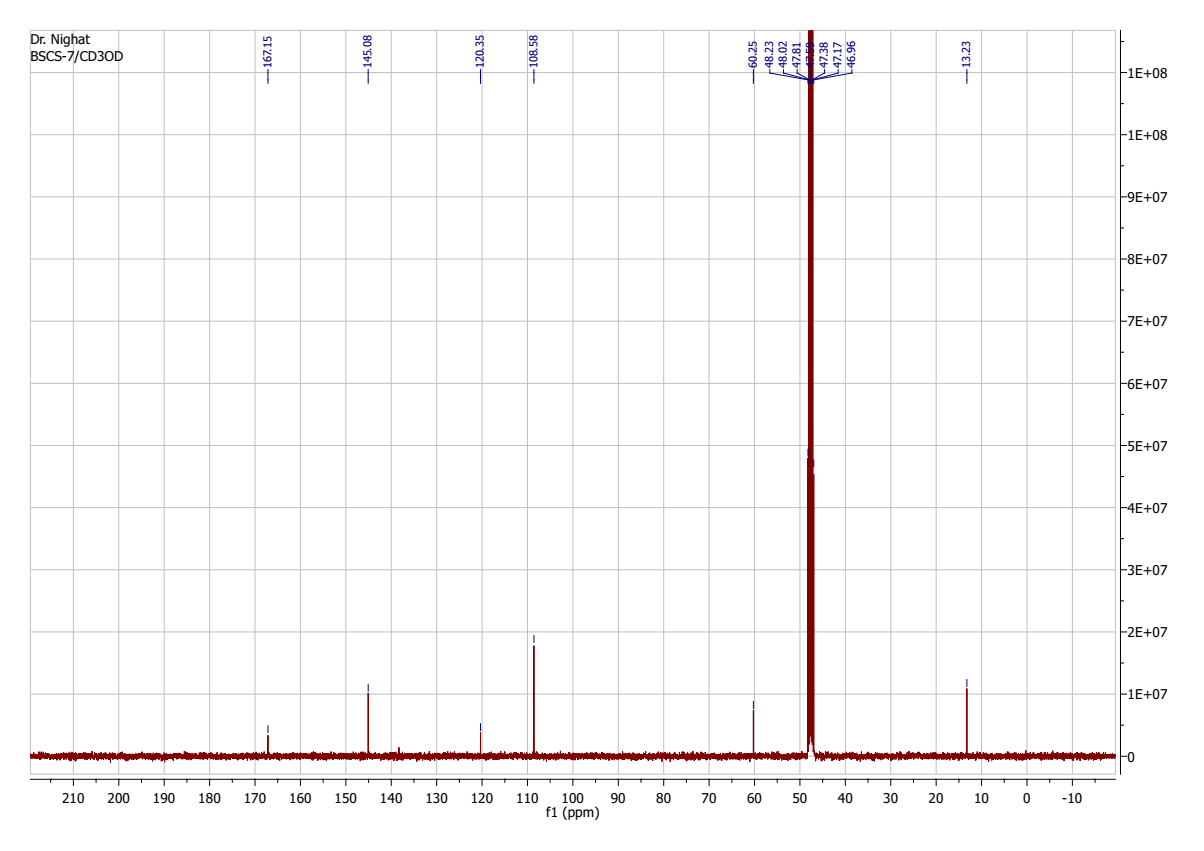
 Figure 20. ^13^C NMR spectrum of compound **3**


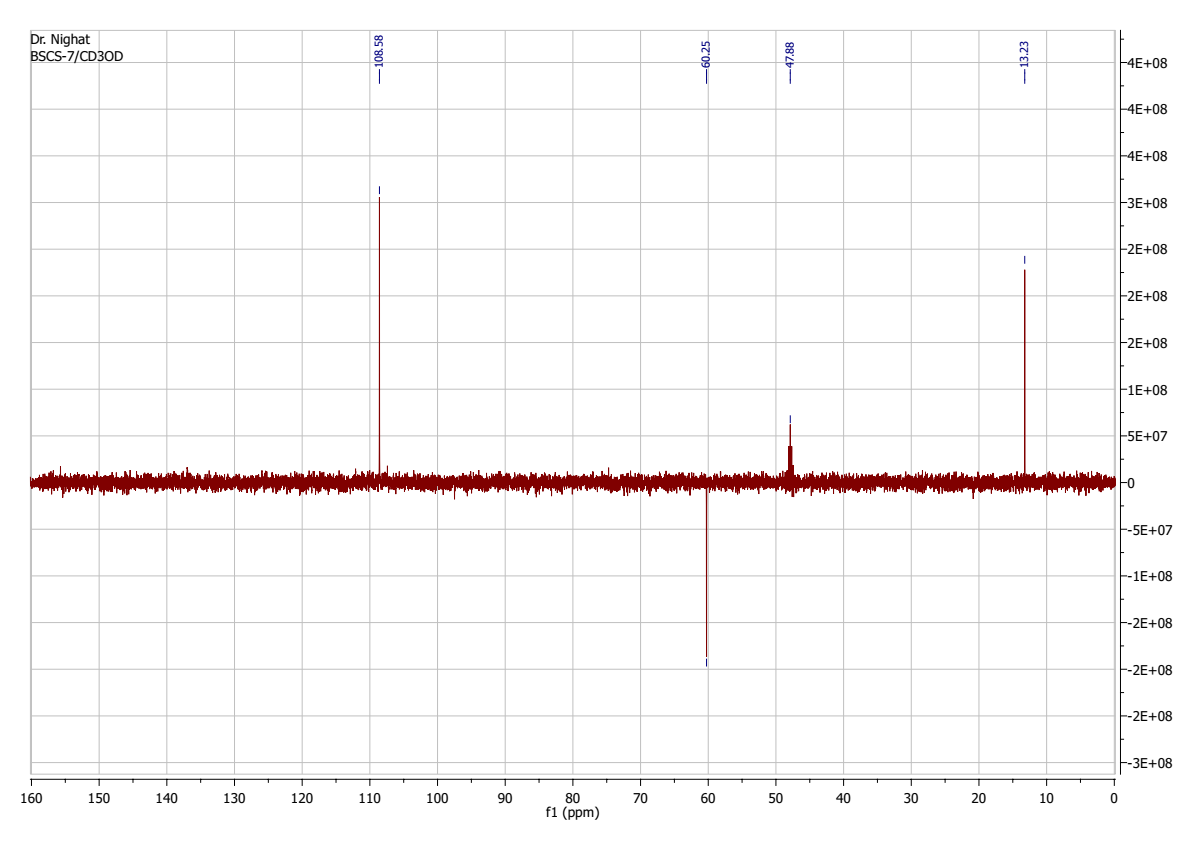
 Figure 21. DEPT 135 spectrum of compound **3**


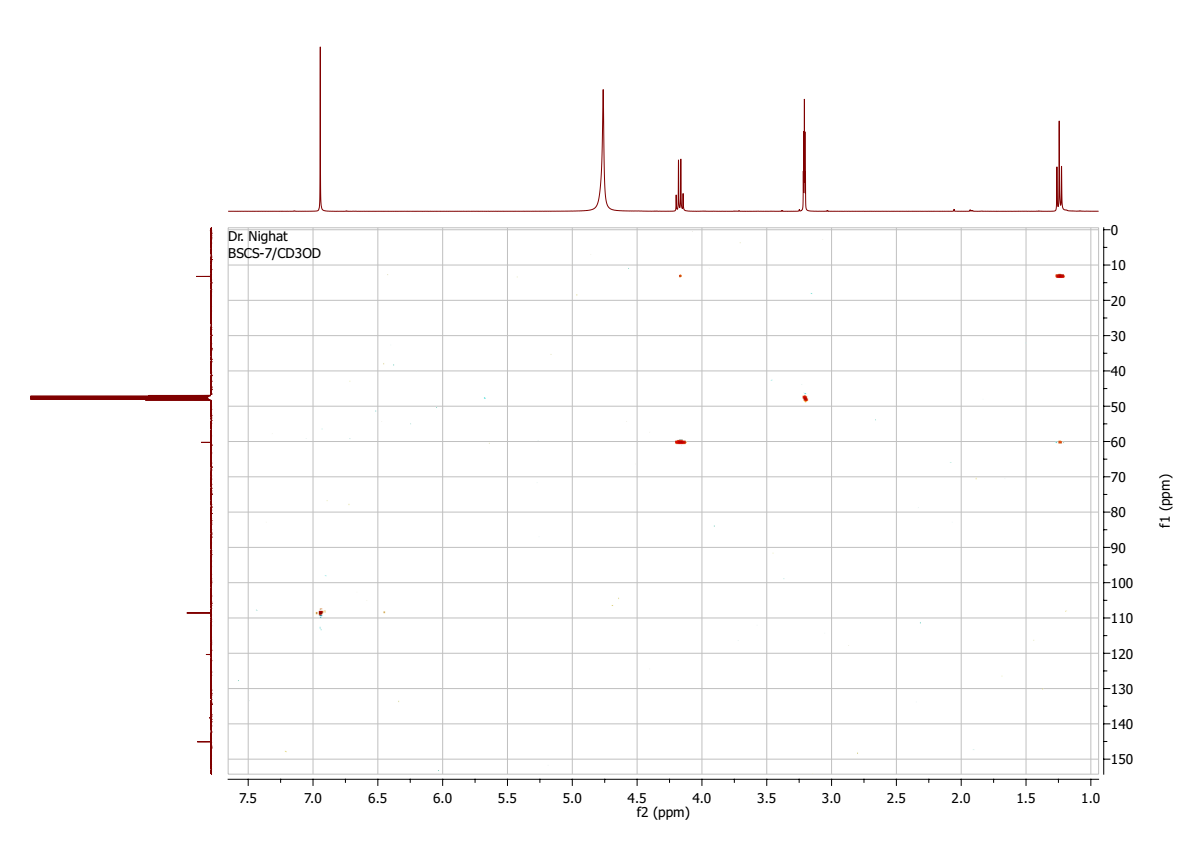
 Figure 22. HSQC spectrum of compound **3**


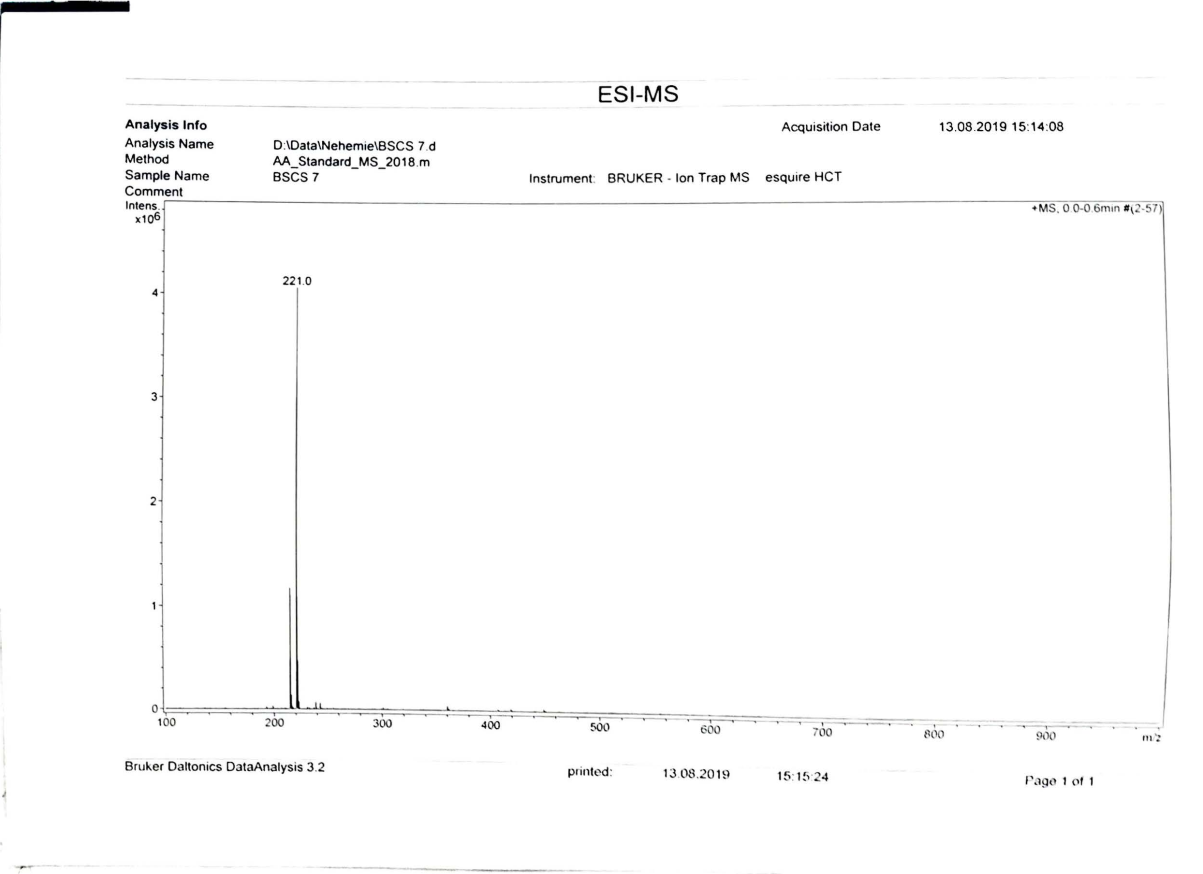
 Figure 23. ESIMS of compound **3**


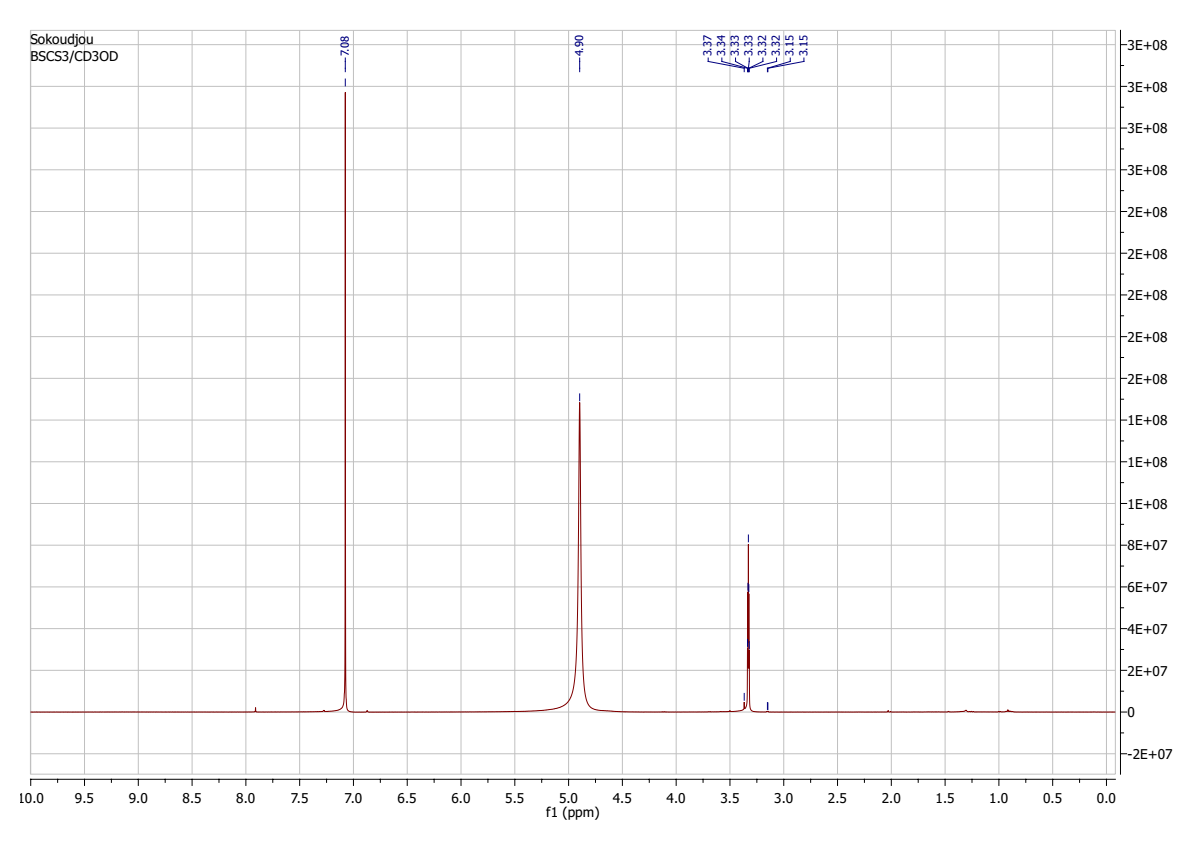
Figure 24. ^1^H NMR spectrum of compound **4**


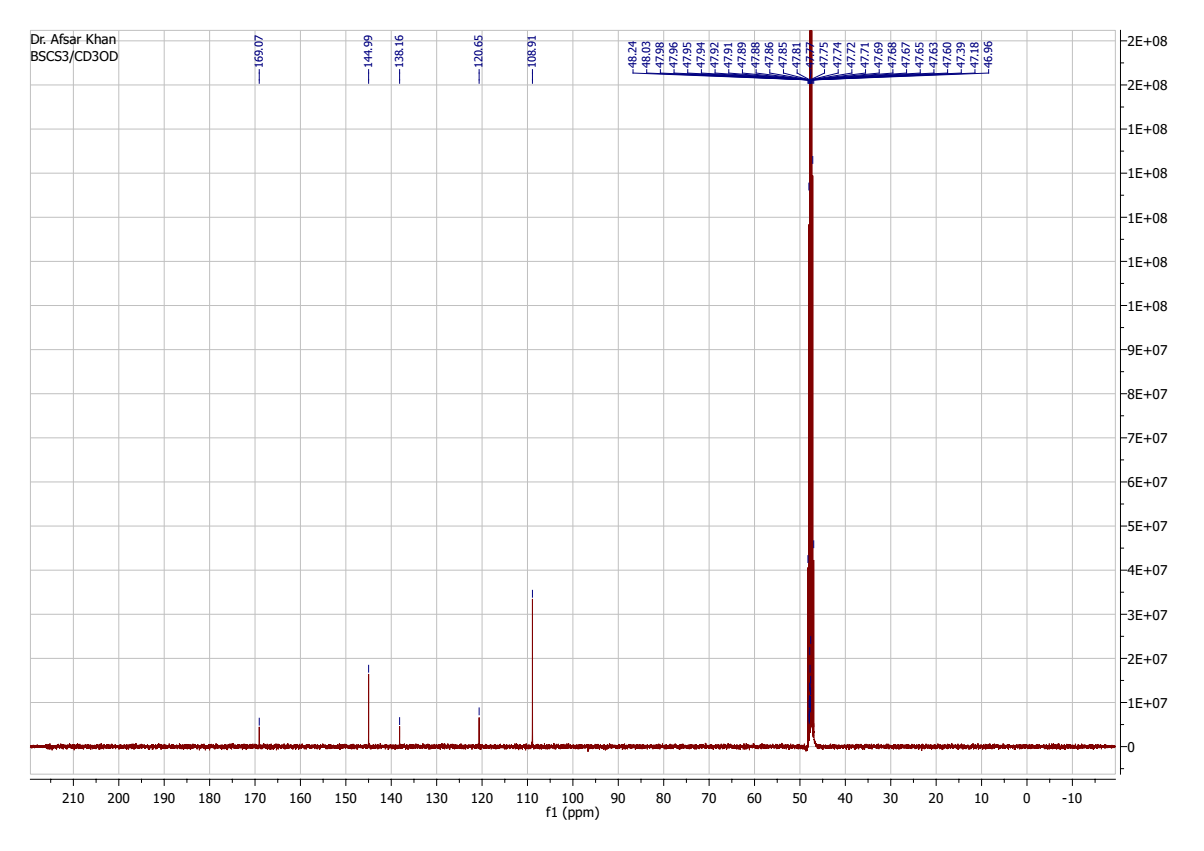
Figure 25. ^13^C NMR spectrum of compound **4**


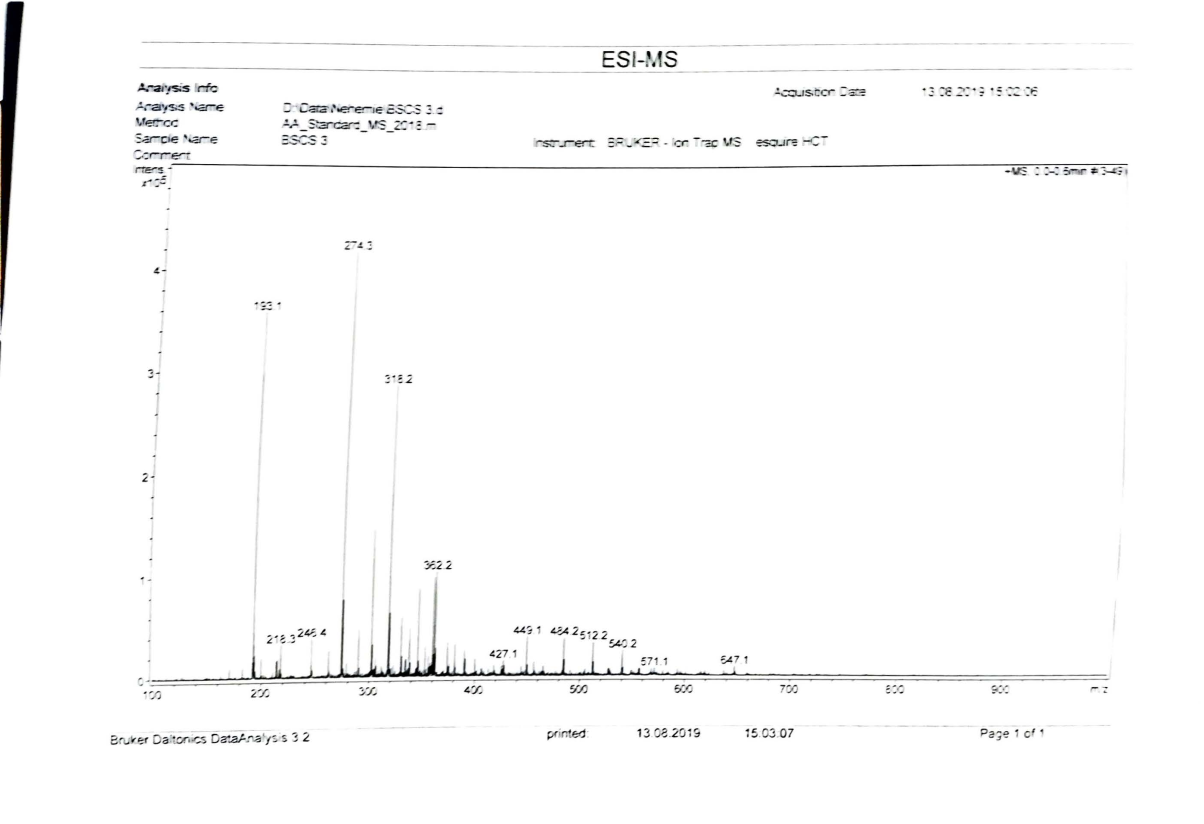


Figure 26. ESIMS of compound **4**
